# Flower and Spikelet Construction in Rapateaceae (Poales)

**DOI:** 10.3389/fpls.2021.813915

**Published:** 2022-01-27

**Authors:** Sofia D. Koblova, Paula J. Rudall, Dmitry D. Sokoloff, Dennis W. Stevenson, Margarita V. Remizowa

**Affiliations:** ^1^Department of Higher Plants, Faculty of Biology, M. V. Lomonosov Moscow State University, Moscow, Russia; ^2^Jodrell Laboratory, Royal Botanic Gardens, Kew, Richmond, United Kingdom; ^3^New York Botanical Garden, Bronx, NY, United States

**Keywords:** Rapateaceae, nectaries, spikelets, gynoecium, floral development, floral anatomy

## Abstract

The family Rapateaceae represents an early-divergent lineage of Poales with biotically pollinated showy flowers. We investigate developmental morphology and anatomy in all three subfamilies and five tribes of Rapateaceae to distinguish between contrasting hypotheses on spikelet morphology and to address questions on the presence of nectaries and gynoecium structure. We support an interpretation of the partial inflorescence (commonly termed spikelet), as a uniaxial system composed of a terminal flower and numerous empty phyllomes. A terminal flower in an inflorescence unit is an autapomorphic feature of Rapateaceae. The gynoecium consists of synascidiate, symplicate, and usually asymplicate zones, with gynoecium formation encompassing congenital and often also postgenital fusions between carpels. Species of Rapateaceae differ in the relative lengths of the gynoecial zones, the presence or absence of postgenital fusion between the carpels and placentation in the ascidiate or plicate carpel zones. In contrast with previous reports, septal nectaries are lacking in all species. The bird-pollinated tribe Schoenocephalieae is characterized by congenital syncarpy; it displays an unusual type of gynoecial (non-septal) nectary represented by a secretory epidermis at the gynoecium base.

## Introduction

Molecular phylogenetic analyses have resulted in expansion of the order Poales to include more than a third of all monocot species (Bouchenak-Khelladi et al., 2014; Hochbach et al., 2018). The order now encompasses *ca* 16 families, including the two major families of wind-pollinated monocots, Poaceae and Cyperaceae, and several smaller families that range from mostly wind-pollinated to predominantly biotically pollinated (Linder and Rudall, 2005; Givnish et al., 2018). However, despite phylogenetic data on Poales from multiple sources, including different genomic markers, transcriptomes, and plastomes (e.g., Bouchenak-Khelladi et al., 2014; Barrett et al., 2016; McKain et al., 2016; Givnish et al., 2018; Hochbach et al., 2018), the precise relationships of some families that lie on very short branches remain problematic.

The family Rapateaceae (Poales) includes 17 genera distributed almost exclusively in South America, though a single monospecific genus, *Maschalocephalus*, occurs in West Africa (Stevenson et al., 1998; Berry, 2004; Givnish et al., 2004). In most molecular phylogenetic analyses of Poales, Rapateaceae are placed in a basal grade with Bromeliaceae and Typhaceae (McKain et al., 2016; Givnish et al., 2018; Hochbach et al., 2018), though rarely they are associated with Mayacaceae within the cyperid clade (Bouchenak-Khelladi et al., 2014). Rapateaceae were traditionally classified into two subfamilies, Rapateoideae and Saxofridericioideae (Stevenson et al., 1998), but this classification was revised by Givnish et al. (2004) as three subfamilies and five tribes (Table 1). Most molecular phylogenetic studies of Poales have used relatively limited sampling within Rapateaceae, making assessment of subfamilial relationships difficult (including plastome sequence data: Givnish et al., 2010, 2018). The most detailed sampling to date involved *ndhF* sequence data (Givnish et al., 2000, 2004), underlining the need for more analyses with greater taxon sampling.

**TABLE 1:** Collection data of species and material examined, arranged according to classification in Givnish et al. (2004).

	Species	Collection data
**Subfamily Rapateoideae**		
	*Cephalostemon gracilis* R.H.Schomb.	K: Sajo, 1998
	*Duckea cyperaceoidea* (Ducke) Maguire	NYBG: Colella 1277
	*D. flava* (Link) Maguire	NYBG: Colella 2068, 2090
	*D. junciformis* Maguire	NYBG: Maguire 35697
	*Rapatea paludosa* Aubl.	NYBG: Colella 1272, DWS 884
	*Spathanthus unilateralis* (Rudge) Desv.	NYBG: Colella 2091, 1744, 2091
**Subfamily Monotremoideae**		
	*Maschalocephalus dinklagei* Gilg & K.Schum.	K: 27251, Adames 469 (Liberia, W. Africa)
	*Monotrema aemulans* Körn.	NYBG: Colella 1276
	*Potarophytum riparium* Sandwith	K: 18097, Sandwith 1382 (Guyana)
**Subfamily Saxofridericioideae**		
**Tribe Saxofridericieae**		
	*Saxofridericia aculeata* Maguire	NYBG: Maas 6920
	*Saxofridericia compressa* Maguire	NYBG: Maguire 42180
**Tribe Stegolepideae**		
	*Stegolepis cardonae* Maguire	NYBG: Pruski 3689
	*Stegolepis* sp.	K: s.n.
**Tribe Schoenocephalieae**		
	*Guacamaya superba* Maguire	NYBG: Colella 1275, Venezuela
	*Kunhardtia rhodantha* Maguire	NYBG: Berry 4808, Colella s.n.
	*Schoenocephalium cucullatum* Maguire	NYBG: Maguire 30486, Colella 1769, 1623

Rapateaceae are well defined by several reproductive characters, especially their showy flowers with large and conspicuous petals and their unusual partial inflorescences, which are often termed spikelets. However, because the Rapateaceae partial inflorescence differs strongly from the grass spikelet, we here use the term reproductive unit (RU). The Rapateaceae RU consists of several empty bracts and a single flower; several RUs are grouped into common head-like inflorescences supported by two involucral leaves. Contrasting hypotheses interpret the single-flowered RU of Rapateaceae as either a uniaxial system with a terminal flower (Trifonova, 1982; Dahlgren et al., 1985; Berry, 2004; Takhtajan, 2009) or a condensed cyme in the form of a bostryx, in which the partial inflorescence is a multiaxial sympodial system and the solitary flower is axillary (Stevenson et al., 1998; Colella-Franco, 1999). More data are needed to evaluate these contrasting hypotheses.

All Rapateaceae are apparently biotically pollinated (Stevenson et al., 1998). However, studies of pollination biology in the family are sparse and sometimes conflicting; a problematic question is whether nectar is produced in some species. Some Rapateaceae apparently offer only a pollen reward via buzz pollination though their poricidal anthers. For example, species of *Rapatea, Saxofridericia* and *Stegolepis* are reportedly exclusively buzz pollinated by female euglossine bees (Renner, 1989; Hentrich, 2008; Krahl et al., 2020). However, intercarpellary slits that resemble septal nectaries were reported in young flowers of *Saxofridericia aculeata* (Ferrari and Oriani, 2017). The presence of septal nectaries is widely inferred for the three genera of Schoenocephalieae (*Guacamaya, Kunhardtia* and *Schoenocephalium*), based on the presence of abundant nectar and visits by hummingbirds (Givnish et al., 2000; Berry, 2004), though pollination is not well-documented in these species (Fernández-Lucero et al., 2016). However, anatomical studies to date have failed to convincingly confirm the presence of septal nectaries in these taxa (Colella-Franco, 1999; Oriani and Scatena, 2013; Ferrari and Oriani, 2017). Venturelli and Bouman (1988) reported open septal nectaries (intercarpellary slits) in the ovary of *Spathanthus unilateralis* (Rapateoideae), but this observation was not confirmed in a subsequent study (Oriani and Scatena, 2013). Septal nectaries have been described in only one other family of Poales, Bromeliaceae, which possesses an inferior ovary with labyrinthine nectaries below the ovary locules (Sajo et al., 2004; Linder and Rudall, 2005).

In this study, we investigate the anatomy and development of floral structures in all three subfamilies of Rapateaceae and evaluate them in the context of other Poales. Although previous studies have examined embryology and anatomy in some Rapateaceae (Colella-Franco, 1999; Oriani and Scatena, 2013), relatively little comparative data exist on floral development and overall gynoecium construction. We discuss the homologies of the partial inflorescence (RU) and propose a new morphological interpretation of the gynoecium in Rapateaceae.

## Materials and Methods

The morphology and anatomy of the inflorescences and flowers were examined in nine species of Rapateaceae from all of the currently designated subfamilies and tribes (Table 1). Floral development was investigated in detail for two species of Rapateoideae: *Duckea flava* and *D. junciformis* and also for *Guacamaya superba* (Schenocephalieae). We also report some aspects of floral development for *Rapatea paludosa* and *Spathanthus unilateralis* (Rapateoideae), *Monotrema aemulans* and *Potarophytum riparium* (Monotremoideae), and *Stegolepis cardonae* (Saxofridericioideae–Stegolepideae). Species examined are listed in Table 1. Material was obtained from voucher specimens deposited at NYBG and in the Spirit Collections of New York Botanical Garden and the Royal Botanic Gardens, Kew (K in Table 1).

Specimens deposited in the Spirit Collections had initially been fixed in FAA, then prepared for long-term storage in 70% ethanol. For scanning electron microscopy (SEM), inflorescences at different developmental stages were dissected in 96% ethanol under an Olympus SZX7 stereomicroscope, dehydrated through absolute acetone, critical-point dried using a Hitachi HCP-2 critical-point drier, then coated with gold and palladium using an Eiko IB-3 ion-coater (Tokyo, Japan). Observations were made using a CAMSCAN S2 SEM (Camscan, Cambridge, United Kingdom) at Moscow University.

Microscope slides deposited in the NYBG and K slide collections were accessed and imaged using a Leitz Diaplan photomicroscope at RBG Kew. For other light microscope observations, mature flowers, and floral buds were sectioned using standard methods of Paraplast embedding and serial sectioning at 15 μm thickness using a Thermo Scientific Microm HM 355s rotary microtome at Moscow University. Sections were stained with Toluidine blue and mounted in Vitrogel mounting medium. Digital photomicrographs were made using a Zeiss Axioplan or Olympus BX53 microscope fitted with digital camera.

## Results

### Organography

The RUs are stalked in *Rapatea paludosa* and *Spathanthus unilateralis* and sessile in the other species examined. Each RU is typically located in the axil of a well-developed subtending bract and is composed of numerous phyllomes and a single flower that is apparently terminal. The phyllomes below the flower are sterile (i.e., they do not subtend flowers).

Flowers are bisexual, trimerous, and pentacyclic. The perianth is biseriate and consists of three green sepals and three showy petals. The sepals are free and alternate with the three uppermost phyllomes; they are of equal size, rigid, upright and sometimes almost indistinguishable in general appearance from the underlying phyllomes at anthesis. As the number of RU phyllomes is not precisely determined, floral orientation is variable with regard to the bract that subtends the RU. However, the most frequent pattern is a flower with the median sepal in the abaxial position and two other sepals in transversal-adaxial positions with respect to the RU-subtending phyllome. The three petals alternate with the sepals; they form a corolla tube that is enclosed by the calyx. The distal regions of the petals are free and each differentiated into a broad region (limb) that is exposed at anthesis. The androecium consists of six stamens in two whorls. The stamens possess long, massive filaments that are basally adnate to the corolla tube. In *Stegolepis*, a stamen tube is formed by the filament bases above their separation from the corolla. The anthers are porous and contain copious pollen.

**FIGURE 1:**
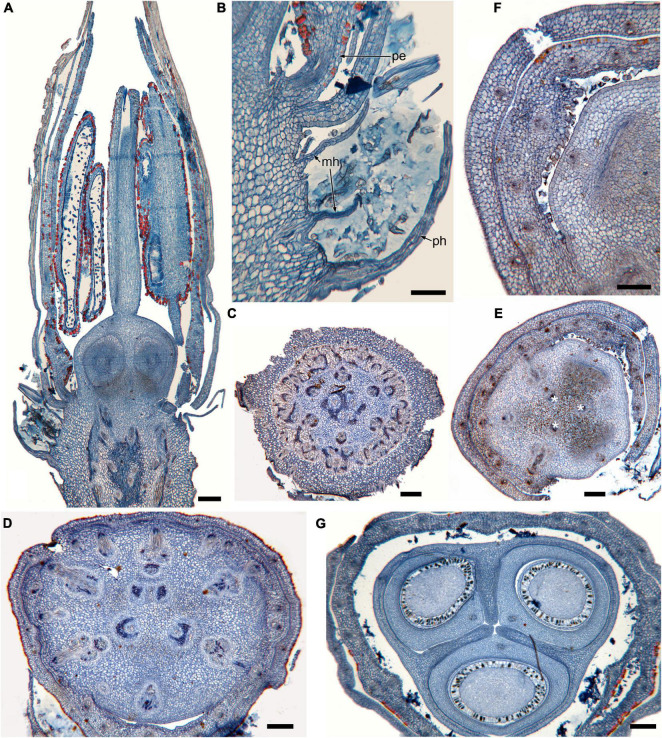
Gynoecium of Rapateoideae. *Cephalostemon gracilis*. **(A,B)** Longitudinal sections of mature flower. **(A)** General view of flower. **(B)** Detail of region between perianth and phyllomes to show mucilage hairs. **(C–G)** Serial cross-sections of mature flower. **(C)** RU axis just below flower. **(D)** Receptacle below gynoecium. **(E)** Synascidiate zone, oblique section, only one of three locules are visible. **(F)** Enlarged portion of panel **(E)** showing secretory hairs around perianth and ovary base. **(G)** Symplicate zone, ovary just above cross-zone. Mh, mucilage-secreting hairs; pe, perianth; ph, phyllomes below flower; *, synventral bundle. Scale bars = 20 mkm in panels **(A,C–E,G)**, 10 mkm in panels **(B,F)**.

**FIGURE 2:**
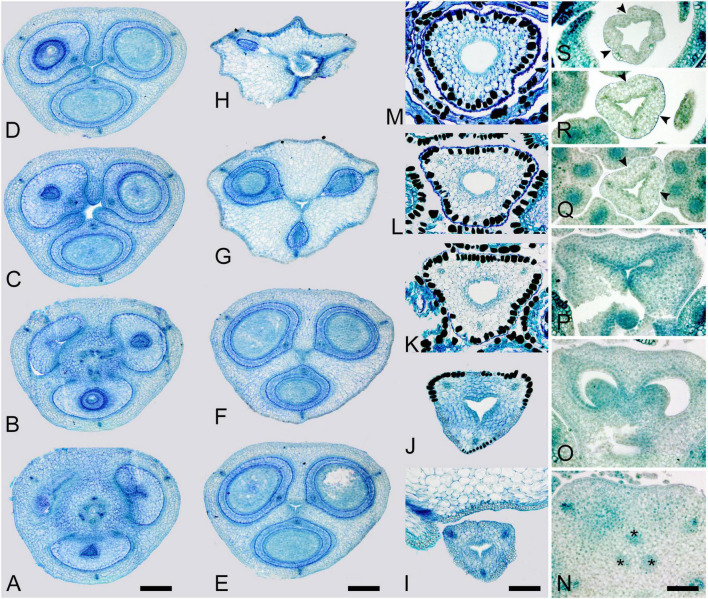
Gynoecium of Rapateoideae. *Duckea junciformis*. **(A–M)** Serial cross-sections through gynoecium of mature flower. **(A)** Synascidiate zone below ovule insertion, ovary. **(B)** Synascidiate zone, level of ovule attachment. **(C,D)** Symplicate zone, ovary just above cross-zone. **(E–G)** Symplicate zone, ovary, note incompletely fused ventral slits to form a canal in gynoecium center. **(H)** Ovary roof, symplicate zone. **(I–M)** Style, possibly asymplicate zone. **(N–S)** Young gynoecium at stage of ovule initiation, serial cross-sections. **(N)** Receptacle below ovary. **(O)** Synascidiate zone, level of ovule insertion. **(P)** Distal part of ovary, symplicate zone. **(Q–S)** Style, asymplicate zone, lines of postgenital fusion between carpels are already weakly discernible at this stage (arrowheads). *, synventral procambial strand. Scale bars = 200 mkm in panels **(A–H)**, 100 mkm in panels **(I–S)**.

**FIGURE 3:**
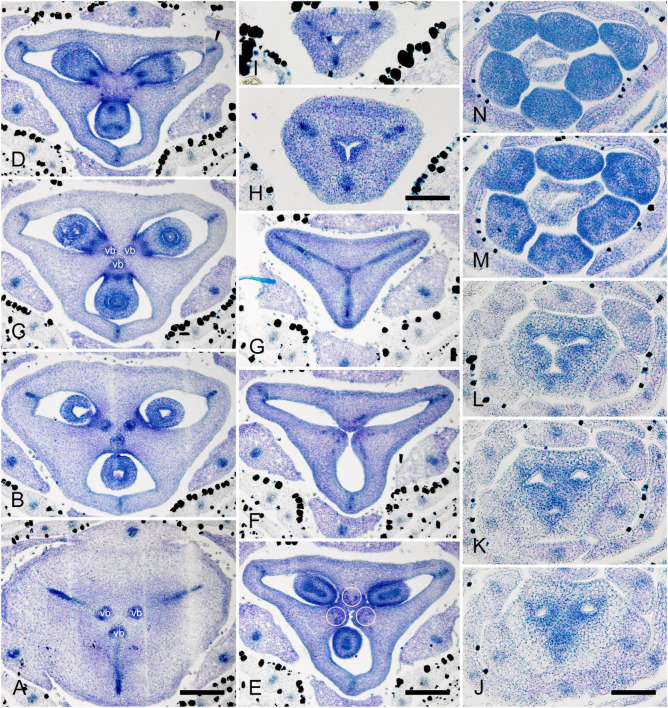
Gynoecium of Monotremoideae. *Maschalocephalus dinklagei.* **(A–I)** Gynoecium of mature flower. **(A)** Receptacle below ovary. **(B)** Basal part of synascidiate zone, ovary base. **(C,D)** Synascidiate zone, level of ovule attachment. **(E,F)** Symplicate zone, distal part of ovary. **(G)** Ovary roof, symplicate zone. **(H)** Style base above ovary roof, symplicate zone. **(I)** Style (distal part), symplicate zone. **(J–N)** Gynoecium of flower bud at stage before ovule initiation. **(J)** Short synascidiate zone, future ovary. **(K)** Transition to symplicate zone, future ovary. **(L–N)** Symplicate zone, future distal ovary and style. Vb, homocarpellous ventral bundle; circle, ventral veins above placenta. Scale bars = 200 mkm in panels **(A–G)**, 100 mkm in panels **(H–J)**.

**FIGURE 4:**
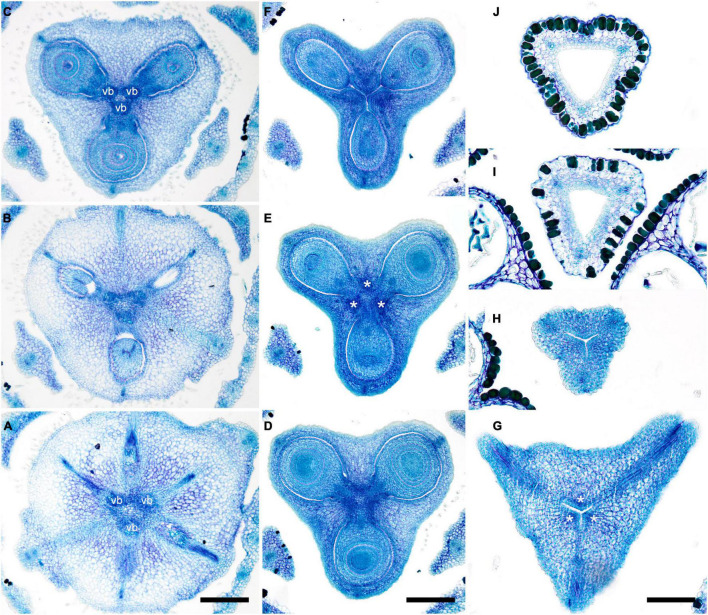
Gynoecium of Monotremoideae. *Potarophytum riparium*, serial cross-sections through mature gynoecium. **(A)** Receptacle below the ovary. **(B)** Basal part of synascidiate zone, ovary base. **(C,D)** Synascidiate zone, level of ovule attachment. **(E)** Transition to symplicate zone. **(F)** Distal part of ovary, symplicate zone. Note open ventral slits. **(G)** Ovary roof with triradial canal in its center, symplicate zone. **(H)** Basal part of style with triradial canal, symplicate zone. **(I)** Style surrounded by stamens, (a)symplicate zone. **(J)** Distal part of style below stigma, level above stamens, asymplicate zone. Vb, homocarpellous ventral bundle; *, synventral bundle. Scale bars = 200 mkm in panels **(A–F)** and 100 mkm in panels **(G–J)**.

**FIGURE 5:**
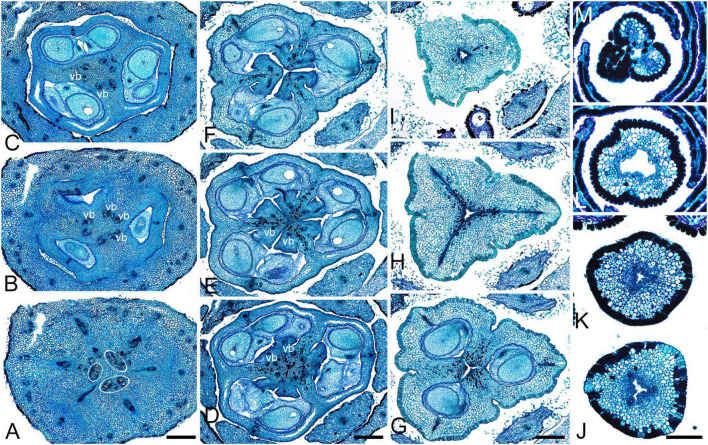
Gynoecium of Saxofridericioideae-Saxofridericieae. *Saxofridericia compressa*, and serial cross-sections through mature flower. **(A)** Receptacle below ovary with three sets of ventral bundles (outlined). **(B,C)** Sterile synascidiate zone, ovary base. **(D–F)** Levels of placentae, symplicate **(D),** and hemisymplicate zone **(E,F)**. **(F)** Unilocular region. **(G–I)** Ovary above placentae and ovary roof, asymplicate zone. Note incompletely closed ventral slits in panel **(G). (J–M)** Style, asymplicate zone. Vb, ventral bundle. Scale bars = 400 mkm in panels **(A–I)** and 200 mkm in panels **(G–M)**.

**FIGURE 6:**
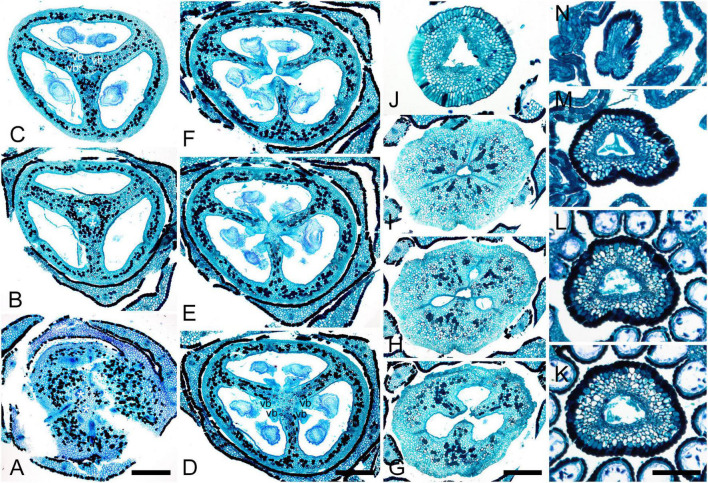
Gynoecium of Saxofridericioideae-Stegolepideae. *Stegolepis cardonae*, serial cross-sections through mature gynoecium. **(A)** Receptacle below ovary. **(B,C)** Sterile synascidiate zone, ovary base. **(D–F)** Levels of placentae. **(D)** Symplicate zone with postgenitally fused ventral slits. **(E)** Transition to unilocular region. **(F)** Hemisymplicate zone, distal part of ovary, unilocular region. **(G–J)** Ovary above placentae and ovary roof, hemisymplicate **(G)**, and asymplicate zones. **(K–N)** Style, asymplicate zone, note postgenitally closed gynoecium tip in panel **(N)**. Note that in this flower median carpel is adaxial in this flower. Vb, homocarpellous ventral bundle; *, heterocarpellous ventral bundle. Scale bars = 400 mkm in panels **(A–J)** and 100 mkm in panels **(K–N)**.

**FIGURE 7:**
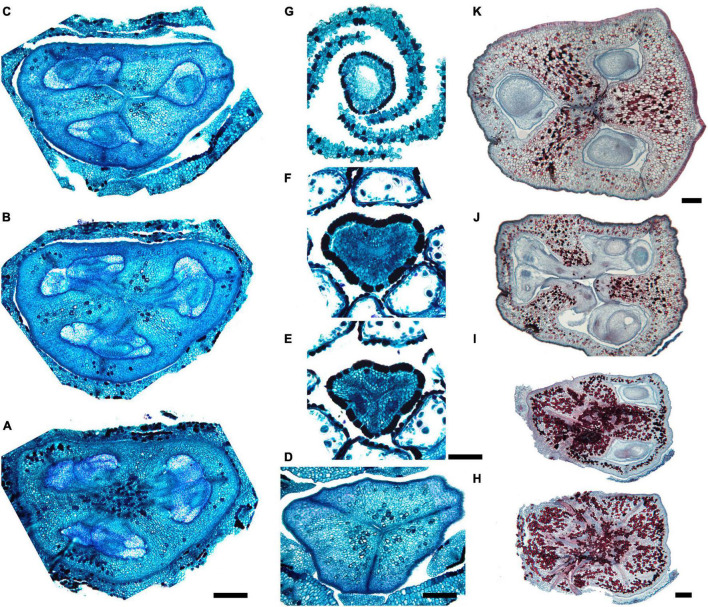
Gynoecium of Saxofridericioideae-Schoenocephalieae. *Guacamaya superba* and *Schoenocephalium cucullatum.* (**A–G)** Serial cross-sections through gynoecium of *Guacamaya superba***. (A)** Basal part of ovary, close to synascidiate zone. **(B)** Middle part of ovary, symplicate zone. **(C)** Distal part of ovary, symplicate zone. Note almost open ventral slits. **(D)** Ovary roof with triradial canal in its center, symplicate zone. **(E,F)** Style with triradial canal filled with mucilage, symplicate zone. **(G)** Stigma (surrounded by contort petals, symplicate zone. **(H–K)** Serial cross-sections through gynoecium *Schoenocephalium cucullatum.* **(H)** Receptacle below ovary. **(I)** Synascidiate zone. Note thin blue-colored epidermis in panels **(H,I)**. **(J)** Symplicate zone, level of placentae. **(K)** Symplicate zone, level above placentae close to ovary roof. Scale bars = 200 mkm in panels **(A–D)**, 100 mkm in panels **(E–G)**, 20 mkm in panels **(H–J),** and 10 mkm in panel **(K)**.

**FIGURE 8:**
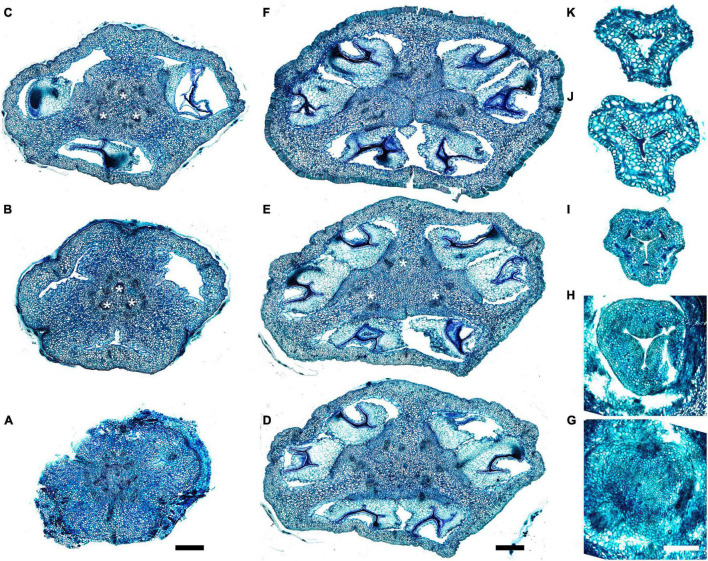
Gynoecium of Saxofridericioideae-Schoenocephalieae. *Kunhardtia rhodantha*. **(A–F)** Ovary of anthetic flower. **(A)** Receptacle below ovary. **(B)** Basal part of synascidiate zone. **(C)** Distal part of synascidiate zone. Note secretion around ovary in panels **(B,C)**. **(D)** Transition to symplicate zone. **(E,F)** Symplicate zone. **(G–K)** Gynoecium of flower bud at stage before ovule initiation. **(G)** Just initiated synascidiate zone, future ovary. **(H,I)** Symplicate zone, future ovary. **(J,K)** Symplicate zone, future style. *, synventral bundle. Scale bars = 400 mkm in panels **(A–F)** and 50 mkm in panel **(G–K)**.

Gynoecial characters of species examined are summarized in Table 2. In all species examined, the ovary is superior and the gynoecium consists of three united carpels and three zones – synascidiate, symplicate and usually asymplicate (Figures 1–8 and Supplementary Figures 1–4) (terminology after Leinfellner, 1950). A hemisymplicate zone is present in *Saxofridericia compressa* (Figure 5) and *Stegolepis cardonae* (Figure 6), in addition to all other zones. Thus, each carpel consists of both ascidiate and plicate zones. In most species examined, the carpels are united partly congenitally and partly postgenitally along their entire length. The ovary is formed by synascidiate and symplicate zones in all genera, i.e., carpels are congenitally united from their inception and develop as an entire structure by zonal growth. The length of the asymplicate zone varies among species examined and in some cases among flowers of the same species. Within the asymplicate zone, the carpels are postgenitally united, i.e., these carpel parts are initiated separately and become fused during their development through contact between previously free epidermal surfaces. In *Saxofridericia* (Figure 5 and Supplementary Figure 3), *Stegolepis* (Figure 6), *Spathanthus* (Supplementary Figures 2A–G) and some flowers of *Duckea* spp. (Figure 2 and Supplementary Figures 1, 2H), the ovary roof and the style are composed of an asymplicate zone with postgenital fusion between carpels. In *Potarophytum* (Figure 4), *Monotrema* and some flowers of *Duckea*, the lower part of the style is composed of a symplicate zone and only the upper part of the style is represented by asymplicate zone. In *Maschalocephalus* (Figure 3) and Schoenocephalieae (Figures 7, 8), the gynoecium seems to be formed without postgenital fusion and an asymplicate zone is absent. Lines of postgenital fusion are indiscernible in mature flowers of Rapateaceae and only weekly discernible in the late buds due to deep tissue redifferentiation, which occurs very early in gynoecium development along the contact areas between the free carpel parts. Thus, to clarify the modes of carpel fusion, we undertook a separate developmental study (below: organogenesis).

**TABLE 2:** Characters of gynoecium morphology and anatomy in Rapateaceae.

Species	Ovule insertion	Ovules per carpel	Bulging locules	Postgenital carpel fusion (asymplicate zone)	Number of locules in (hemi)symplicate zone (ovary)	Stylar canal closed above ovary roof	Tannins in ovary parenchyma	Synventral bundles	Ventral bundles present above ovules
**Subfamily Rapateoideae**									
*Cephalostemon gracilis* (Figure 1)	Ascidiate zone	1	−	?	3	+	+ below ovary locules and synascidiate zone	+	−
*Duckea flava* (Supplementary Figures 1, 2H, 5, 6)	Ascidiate zone	1	+	+(variable length)	3	+	−	+ below ovary locules	−
*Duckea junciformis* (Figures 2, 9, 10)	Ascidiate zone	1	+	+(variable length)	3	−	−	+ below ovary locules	−
*Rapatea paludosa* (Supplementary Figures 2I–L)	Ascidiate zone	1	+	+(upper part of style)	3		+	+	−
*Rapatea paludosa* (Figures 11G,H)	Ascidiate zone	1	+	+(upper part of style)	3		+ below ovary locules and synascidiate zone	+	−
*Spathanthus unilateralis* (Figures 11A–F and Supplementary Figures 2A–G)	Ascidiate zone	2	++	+(ovary roof and style)	3	−	−	+	+
**Subfamily Monotremoideae**									
*Maschalocephalus dinklagei* (Figure 3)	Ascidiate zone	1	−	−	3	+	−	−	+
*Monotrema aemulans* (Figures 12A–D)	Ascidiate zone	1	−	+(upper part of style)	3		+ below ovary locules and synascidiate zone	+ in symplicate zone (ovary)	+
*Potarophytum riparium* (Figures 4, 12E–G)	Ascidiate zone	1	−	+(upper part of style)	3	−	−	+ in symplicate zone (ovary)	+
**Subfamily Saxofridericioideae**									
**Tribe Saxofridericieae**									
*Saxofridericia aculeata* (Supplementary Figure 3)	Plicate zone	Several	−	+(ovary roof and style)	1	−	+ below ovary locules and synascidiate zone	+ ovary	+
*Saxofridericia compressa* (Figure 5)	Plicate zone	Several	−	+(ovary roof and style)	1	−	+ septae	−	−
**Tribe Stegolepideae**									
*Stegolepis cardonae* (Figures 6, 13)	Plicate zone	Several	−	+(ovary roof and style)	1	−	+ ovary wall and septae	+ synascidiate zone (ovary)	
**Tribe Schoenocephalieae**									
*Guacamaya superba* (Figures 7A–G, 14 and Supplementary Figure 4)	Plicate zone	Several	−	−	3	+	+ ovary wall and septae	+ synascidiate zone and below ovary locules	−
*Kunhardtia rhodantha* (Figure 8)	Plicate zone	Several	−	−	3	+	+ ovary wall and septae	+ ovary	−
*Schoenocephalium cucullatum* (Figures 7H–K)	Plicate zone	Several	−	−	3	−	+ ovary wall and septae	+ ovary	−

Ventral slits in the plicate zone are either open or postgenitally closed. Within the ovary, the ventral slits are almost completely postgenitally closed in the plicate zone in representatives of Rapateoideae (Figures 1, 2), Monotremoideae (Figures 3, 4), and Schoenocephalieae (Figures 7, 8). The ovary is trilocular throughout its entire length in Schoenocephalieae. In Rapateoideae (i.e., *Duckea, Rapatea*, and *Spathanthus*) and Monotremoideae (*Potarophytum* and *Maschalocephalus*) a very short unilocular region is usually present just above the cross-zone. In the tribes Saxofridericieae (*Saxofridericia*, Figure 5 and Supplementary Figure 3) and Stegolepidaeae (*Stegolepis*, Figure 6), the ovary is unilocular above the cross-zone almost up to the ovary roof. The ovules are anatropous. The common style has a single stylar canal that continues basally into the ovary in most species examined, or is closed at the base. In most species examined, the stylar canal is closed at its tip via postgenital fusion. In *Maschalocephalus* (Figure 3) and Schoenocephalieae (Figures 7, 8), the stylar canal opens apically in the stigmatic region, where it is sealed by secretion.

The relative lengths of the gynoecial zones and the locations of the placentae differ between subfamilies. The synascidiate zone was fertile in species examined of the subfamilies Rapateoideae (Figures 1, 2 and Supplementary Figures 1, 2) and Monotremoideae (Figures 3, 4). All Rapateoideae except *Spathanthus* possess a single ovule per carpel attached to a cross-zone. In *Spathanthus* (Supplementary Figures 2A–G), there are two ovules per carpel inserted side by side at slightly different levels. In both subfamilies, the micropyle is oriented toward the ovary base and the chalazal side of the ovule is located within the distal part of the ovary. The ovary locules are bulging and the style is somewhat gynobasic in some Rapateoideae (*Duckea junciformis*, *D. flava, Spathanthus*, and *Rapatea paludosa*) and Saxofridericioideae (*Saxofridericia aculeata*). Although the ovules are inserted in the cross-zone, they are mainly located in the plicate carpel zone.

In all Saxofridericioideae examined, placentae with numerous ovules are located in the plicate carpel zone, whereas the ascidiate zone is sterile (Figures 5–8 and Supplementary Figures 3, 4). The micropyles are oriented toward the ovary wall. The ovary is composed of synascidiate and symplicate zones in *Saxofridericia aculeata* and all Schoenocephalieae, and synascidiate, symplicate and hemisymplicate zones in *Stegolepis* and *Saxofridericia compressa*.

Although small septal slits (resembling non-internalized septal nectaries) were observed here in *Spathanthus* (Supplementary Figures 2A–G), distinct septal (gynopleural) nectaries are absent from all species examined, including representatives of the bird-pollinated tribe Schoenocephalieae, in which nectar secretion has been observed. Younger flowers of *Duckea* (Rapateoideae) show non-secretory cavities in the septae, but such cavities are absent from mature flowers. Schizogenous air spaces are present within the ovary wall in some species (e.g., *Duckea*). We also observed a deeply sunken region around the top of the style in *Duckea flava* (Supplementary Figure 2H) and other species with a gynobasic style, which could function as a nectar source. In the absence of specific field-based studies using fresh material, our observations cannot conclusively demonstrate the presence of nectaries. One problem is the presence of mucilage hairs in many species; for example, in *Cephalostemon* (Figure 1), long biseriate mucilage-secreting hairs are present on the axis at the petal bases and above the outer phyllomes. In Schoenocephalieae, nectar is apparently secreted from the epidermis at the ovary base; this region lacks mucilage hairs, and the epidermal cells are relatively dark-staining and thin-walled, compared with thickened cells in the upper parts of the ovary (Figures 7H,I, 8B,C).

### Organogenesis

#### Rapateoideae (*Duckea* spp.)

**FIGURE 9:**
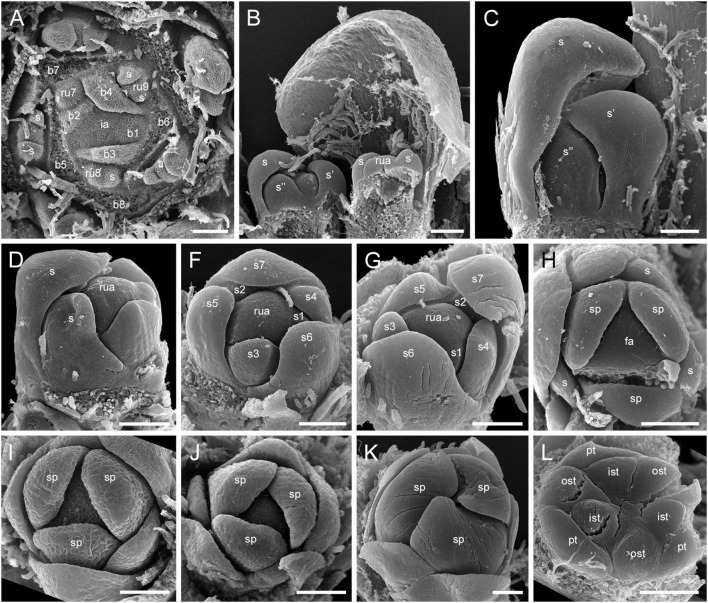
Flower development in Rapateoideae. *Duckea junciformis*, early flower development. **(A)** Inflorescence tip with initiating RUs (“spikelets”). RUs appear in axils of RU-subtending bracts. The first visible RU is present in the axil of seventh youngest bract. Bracts are numbered starting from inflorescence apex. Numbers of RUs correspond to their subtending bracts. Phyllomes on the RU axis first appear in RU located in the axil of 8^th^ bract. Phyllomes are labeled in the order of their initiation (s, first; s’, second; s”, third) **(B,C)** Young RUs with spirally initiating phyllomes, viewed from adaxial side. Phyllomes are labeled in the order of their initiation (s, first; s’, second; s”, third). **(D–G)** Later RUs with spirally initiating phyllomes, numbered starting from RU apex. **(H)** RU terminated by a flower with sepals initiated. Note that sepals alternate with the uppermost phyllomes, which are smaller than the sepals. Sepal estivation is valvate at this stage. **(I,K)** Subsequent stages of calyx enlargement. Sepals grow and their estivation becomes contort. **(L)** Initiation of petals and androecium. Sepals removed. B, RU-subtending bract; fa, floral apex; ia, inflorescence apex, ist, inner stamen; ost, outer stamens; pt, petals; rua, RU apex; s, phyllomes on RU axis; sp., sepals. Scale bars = 100 mkm.

The RUs (spikelets) are initiated acropetally in the axils of spirally arranged bracts (Figure 9A and Supplementary Figure 5A). RU primordia are transversally elongated and smaller than the space available in the axil of the subtending bract. On the RU axis, the first organs initiated are two transversal phyllomes, which appear sequentially (Figures 9A,B and Supplementary Figure 5A). In our material, the first phyllome was always initiated on the right side of the RU meristem in *D. flava* and on the left side in *D. junciformis*. The second phyllome is initiated strictly opposite the first one. From this point onward, the RU meristem is rounded and convex. The third phyllome arises adaxially (Figures 9B,C and Supplementary Figure 5A). All subsequent phyllomes arise in a spiral sequence with a divergence angle that is close to 135° (Figures 9D–G and Supplementary Figures 5B,C). There are no vestigial buds or flowers in the axils of the RU phyllomes.

After all phyllomes are initiated, the RU meristem produces a terminal flower (Figure 9H and Supplementary Figure 5D). There is no evidence that the flower belongs to the axil of any of the uppermost phyllomes. A young flower with only the sepals visible is easily recognizable due to the slightly different shape of the sepals and the triangular outline of the floral meristem after sepal initiation. The RU phyllomes immediately below the flower are smaller than the sepals before the corolla is initiated (Figure 9H and Supplementary Figure 5D). The sepal primordia arise simultaneously and are of equal size and shape. There is apparently a long plastochron after calyx inception. After calyx inception, the floral meristem is triangular as well as each of the young sepals. The sepals grow rapidly; they possess considerably broader bases than the petals and stamens and collectively occupy the entire circumference of the floral bud, serving as protective organs (Figures 9I,K and Supplementary Figure 5E).

Above the calyx, the petal primordia and all stamen primordia appear simultaneously (Figure 9L and Supplementary Figures 5F–H). The bar-shaped petal primordia alternate with the sepals, and two whorls of rounded stamen primordia become visible opposite the sepals and petals. The outer stamen primordia are larger than the inner ones. After petal and stamen initiation, the floral meristem appears almost exhausted and time is needed to produce enough space for the gynoecium.

**FIGURE 10:**
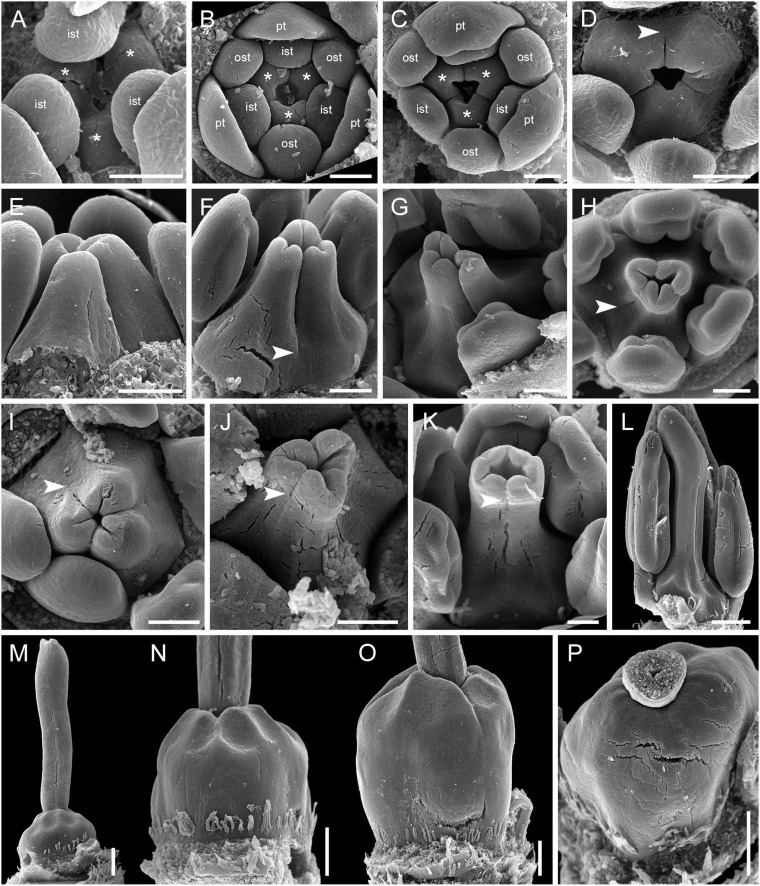
Flower development in Rapateoideae. *Duckea junciformis*, gynoecium development. **(A)** Gynoecium initiation as three separate carpel primordia. **(B)** Very young plicate carpels are united via zonal growth. **(C,D)** Gynoecium with prominent asymplicate zone. Elongation of free plicate carpel parts (asymplicate zone); surrounding organs removed in panel **(D)** to show gynoecium base. **(E–G)** Gynoecium with prominent asymplicate zone. Further elongation of free plicate carpel parts (asymplicate zone). **(H)** Gynoecium with prominent asymplicate zone. Beginning of postgenital fusion between the carpels. Lines of postgenital fusion are clearly visible. **(I)** Gynoecium with short asymplicate zone. Stage similar to panel **(E)**. **(J,K)** Boundaries between the carpels are still tracable. Ovary becomes separated from the style. Style tip showing stylar canal in panel **K**. **(L)** Lines of postgenital fusion are no longer visible starting from this stage. **(M–O)** Progressive stages of ovary elongation. **(P)** Mature ovary with style removed to show stylar canal. *, carpels; ist, inner stamen; ost, outer stamens; pt, petals. Arrowheads show visible boundaries of postgenital fusion in asymplicate zone. Scale bars = 100 mm in panels **(A–K)** and 300 mkm in panels **(L–P)**.

The carpels appear simultaneously as three free rounded carpel primordia on the slightly concave receptacle (Figure 10A and Supplementary Figures 5I, 6A). A continuous ring uniting the peripheral edges of the carpels is present at the stage when the carpel margins become visible (Figure 10B and Supplementary Figure 6B). In some flowers, this rim is more prominent. The free carpel regions (asymplicate zone) elongate and fuse postgenitally to form the style and ovary roof (Figures 10C–H and Supplementary Figures 6C–G). Carpel fusion proceeds from the style to the ovary roof and from inside the gynoecium toward the periphery in *D. junciformis*. The process of postgenital fusion starts early in gynoecium development, prior to ovary formation, which develops by intercalary zonal growth. The style elongates considerably after postgenital fusion is completed and all the boundaries between the carpels have vanished (Figures 10L–P). In some young flowers, we found slits similar to a septal nectary opening close to the ovary roof, but these disappear further into development. The ovary enlarges as an entire structure via zonal growth below the asymplicate zone. Within the ovary, the synascidiate zone with placentae is the last to be initiated (Figures 2N–S). Postgenital closure of the ventral slits occurs slightly later than postgenital fusion between the carpels.

In. *D. flava*, postgenital fusion between the carpels proceeds in an acropetal direction. In this species, the lines of postgenital fusion are no longer visible when carpels are slightly longer than the stamens (Supplementary Figures 6I–L). Some flowers of both species of *Duckea* almost lack any signs of postgenital fusion between the carpels (Figures 10I,K and Supplementary Figure 6H). In such cases, the gynoecium is predominantly formed by congenital fusion and the asymplicate zone is very short.

**FIGURE 11:**
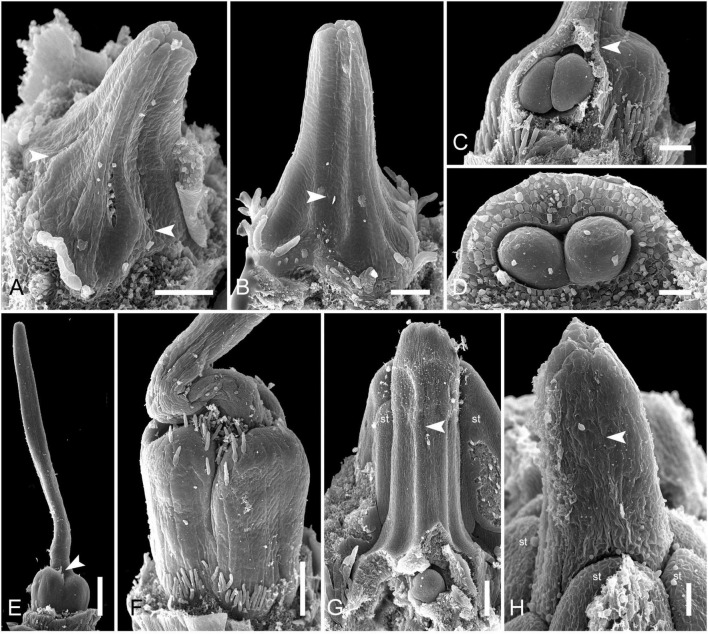
Flower development in Rapateoideae. *Spathanthus unilateralis* and *Rapatea paludosa*, late stages of gynoecium development. **(A–F)** *Spathanthus unilateralis*. **(A,B)** Subsequent stages of relatively young gynoecia with well differentiated ovary. Lines of postgenital fusion are clearly visible. Note that ovary locules are not bulging at these stages and carpel tips form triradiate pattern which is needed to complete postgenital gynoecium closure. **(C,D)** Dissected ovaries with slightly bulging locules to show two pendent ovules per carpel. Side view **(C)** and view from ovary bottom **(D)**. Gynoecium with prominent asymplicate zone. Elongation of free plicate carpel parts (asymplicate zone), surrounding organs are removed in panel **(D)** to show gynoecium base. **(E)** Young gynoecium with postgenitally closed style. **(F)** Mature ovary with bulging locules. **(G,H)** *Rapatea paludosa*. **(G)** Gynoecium at stage of ovule initiation **(H)** Style tip, stage later than in panel **(G)**. St, stamen. Arrowheads show visible boundaries of postgenital fusion in asymplicate zone. Scale bars = 100 mkm in panels **(A–C,G)**, 50 mkm in panel **(D)**, 500 mkm in panel **(E)**, 300 mkm in panel **(F)**, and 30 mkm in panel **(H)**.

In late gynoecia of *Spathanthus* (Figures 11A–E), lines of postgenital fusion between carpels are visible at the ovary roof and along the entire style length. The characteristic bulging locules of *Spathanthus* appear very late in gynoecium development, and small septal slits are visible (Figures 11E,F and Supplementary Figures 2A–G). In floral buds of *Rapatea*, lines of postgenital fusion are visible at least in the upper part of the style (Figures 11G,H).

### Monotremoideae (*Monotrema aemulans* and *Potarophytum riparium*)

**FIGURE 12:**
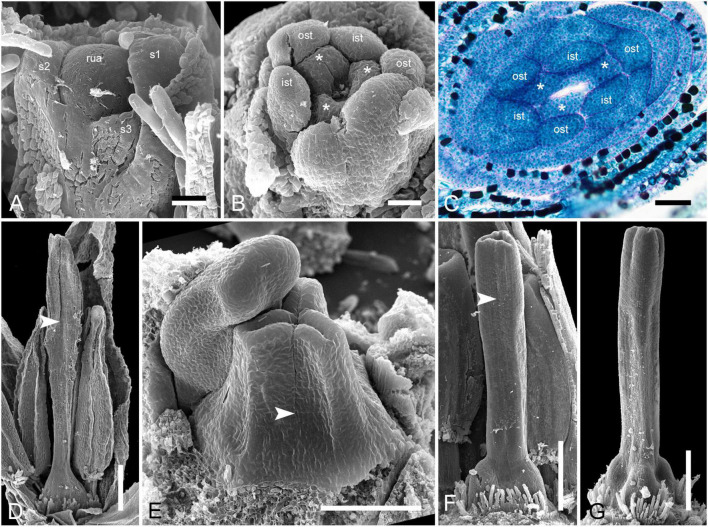
Flower development in Rapateoideae. *Monotrema aemulans* and *Potarophytum riparium*. **(A–D)** Floral development in *Monotrema aemulans* **(A)** Young RU with spirally initiating phyllomes, viewed from adaxial side. Phyllomes are labeled in the order of their initiation. **(B)** Very young nearly free plicate carpels. **(C)** Cross-section of flower at stage as in panel **(B)** to show that carpels are basally connate. **(D)** Late gynoecium. Lines of postgenital fusion in the upper third of style. **(E–G)** Late gynoecium development in *Potarophytum riparium*. **(E)** Young gynoecium with clearly visible lines of postgenital fusion. **(F,G)** Late gynoecia with weakly visible lines of postgenital fusion. Arrowheads show visible boundaries of postgenital fusion in asymplicate zone. ist, inner stamen; ost, outer stamens; rua, RU apex; s, phyllomes on RU axis; *, carpels. Scale bars = 30 mkm in panels **(A,B)**, 50 mkm in panels **(C,E)**, and 300 mkm in panels **(D,F,G)**.

In *Monotrema aemulans*, early stages of RU development show the same order of phyllome initiation as in *Duckea* (Figure 12A). The carpels are initiated by separate primordia but become connected very early (Figures 12B,C). In mature gynoecium, the asymplicate zone is confined to the upper third of the style (Figure 12D). In *Potarophytum riparium*, we were able to trace only late stages of gynoecium development. The carpels are initially free in the upper part of the style (Figure 12E). As in the other genera, the style elongates considerably before the ovary is completely differentiated. Lines of postgenital fusion can be traced in the upper third of the style when the ovary is relatively small (Figures 12F,G).

### Saxofridericioideae–Stegolepidaeae (*Stegolepis cardonae*)

**FIGURE 13:**
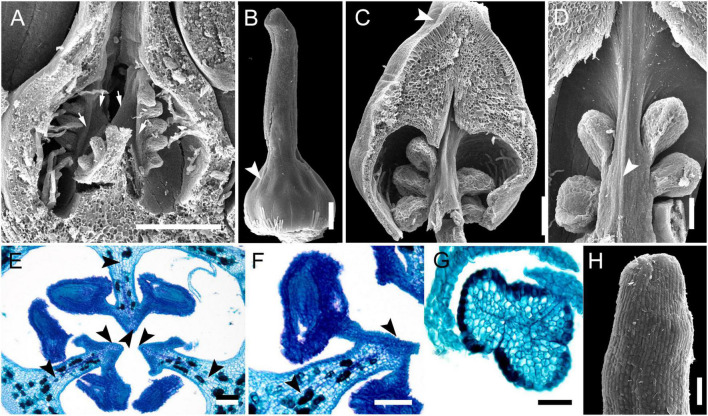
Gynoecium development in Saxofridericioideae-Stegolepideae. *Stegolepis cardonae*, ovary development. **(A)** Young ovary dissected to show placentae in hemisymplicate zone, four of six carpel margins are visible (arrows). **(B)** Gynoecium at stage as in panel **(A)**. Lines of postgenital fusion can be traced from stigma down to ovary roof. **(C)** Later stage with postgenotally fused carpel margins in hemisymplicate zone. Note massive ovary roof. **(D)** Same stage as in panel **(C)**, boundary between two carpels, placenta. **(E)** Cross-section of ovary through hemisymplicate zone, stage similar to **(C,D)**. Incomplete septae are partly formed by postgenital fusion between initially free carpel margins. **(F)** Boundary between two carpels, slightly above **(E)**. **(G)** Section through postgenitally closed gynoecium tip, slightly below stigma. **(H)** Style tip. Arrowheads show visible boundaries and lines of postgenital fusion in asymplicate and hemisymplicate zones. Scale bars = 300 mkm in panels **(A–D)**, 100 mkm in panels **(E,F,H)**, and 50 mkm in panel **(G)**.

The most remarkable feature of *Stegolepis* is the presence of a hemisymplicate zone, which becomes evident in young ovaries (Figure 13A). Here, the carpel margins are free toward the ovary center. In younger ovaries, these carpel margins are gaping but later they meet and fuse postgenitally. Lines of postgenital fusion inside septae can still be traced in late buds (Figures 13C–F). Postgenital fusion between carpels is present from the level of the ovary roof (Figures 13B,G,H).

### Saxofridericioideae–Schoenocephalieae (*Guacamaya superba*)

**FIGURE 14:**
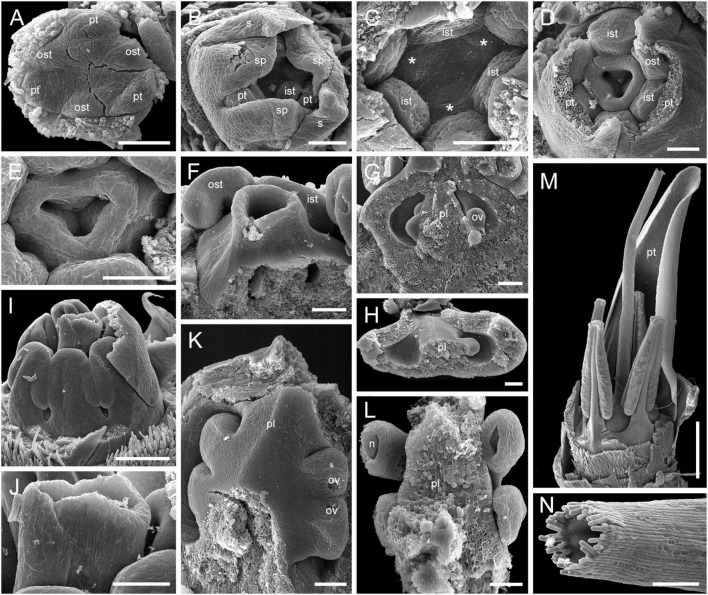
Flower development in Saxofridericioideae-Schoenocephalieae. *Guacamaya superba*. **(A)** Initiation of petals and androecium. Sepals removed. **(B)** General view of flower at gynoecium initiation. **(C)** Gynoecium initiation as three separate carpel primordia. **(D,E)** Young gynoecium with prominent symplicate zone with placentae developing inside the ovary. **(F)** Gynoecium with partly dissected ovary showing very young ovules inside. **(G,H)** Stage similar to panel **(F)**. Dissected ovaries with placentae and young ovules. There are three parietal placentae formed by congenitally fused carpel margins at this stage. Symplicate zone. **(I)** General view of flower showing androecial tube. **(J)** Detail of panel **(I)**. Style with carpel tips. Note that carpels are congenitally united and there no lines of postgenital carpel fusion. **(K,L)** Isolated placenta (symplicate zone), stage similar to panel **(I)**. Views from gynoecium center **(K)** and from the ovary wall **(L)**. **(M)** Nearly mature flower with perianth removed. **(N)** Close up of the style tip in panel **(M)**. *, carpel tips; ist, inner stamen; ost, outer stamens; n, nucellus; ov, ovule; pl, placenta; pt, petals; s, phyllomes on RU axis; sp., sepals. Scale bars = 100 mkm in panels **(A–H)**,**(K,L)**,**(N)**, 300 mkm in panel **(I)** and 1000 mkm in panel **(M)**.

We studied gynoecium development in *G. superba*, in which earlier stages of flower development resemble those described above for *Duckea* (Figures 14A–C). The gynoecium appears as a triangular rim with three more or less pronounced bulges on the radii of the sepals (Figures 14C–E). The gynoecium develops as an entire structure by zonal growth. The symplicate zone (style and most of the ovary) is the first to appear. The sterile synascidiate zone (basal part of the ovary) differentiates at later stages (Figures 14F–L). Each of the three placentae are initially parietal and the ovary is unilocular in the symplicate zone (Figures 14G,H,K). During ovule formation, the placentae meet in the gynoecium center and the ovary becomes trilocular due to postgenital closure of the ventral slits. The gynoecium remains open at its tip (Figures 14M,N).

## Discussion

### Spikelet Homologies

Spikelets of contrasting morphology represent a highly characteristic feature of Poales, especially the wind-pollinated families Poaceae, Cyperaceae and Restionaceae (Linder and Rudall, 2005). A spikelet is a racemose (partial) inflorescence, a uniaxial system composed of an axis bearing several phyllomes, with the flowers located in their axils. In most Poales, the term “spikelet” is used instead of “spike” to highlight the arrangement of small spikelets in complex inflorescences. Typical spikelets of Poales lack a terminal flower (Eiten, 1976; Vrijdaghs et al., 2010; Kellogg, 2015), unless the problematic reproductive structures of Cyperaceae–Mapanioideae are interpreted as partial inflorescences (Richards et al., 2006; Vrijdaghs et al., 2006; Prychid and Bruhl, 2013; Monteiro et al., 2016). In the biotically pollinated families Bromeliaceae, Eriocaulaceae, and Xyridaceae, the inflorescences are racemose and lack a terminal flower (Stützel, 1982; De Sousa et al., 2008; Remizowa et al., 2012; Nogueira et al., 2017, 2021). In Mayacaceae, the flowers are solitary and axillary (Oriani and Scatena, 2019).

The RUs of Rapateaceae are traditionally termed spikelets but they demonstrate a contrasting morphology, with sterile phyllomes on the axis and a single flower that is apparently terminal. Interpreting any single-flowered inflorescence as a spike or spikelet (rather than a capitulum) requires placement of the taxon in its phylogenetic context (Sokoloff and Remizowa, 2021). Several hypotheses have been proposed to relate the Rapateaceae RUs with the spikelets of their presumed close relatives, focusing on branching mode (monopodial vs. sympodial), flower position (true terminal vs. lateral), and the nature of the RU phyllomes.

Our developmental data support the interpretation of the Rapateaceae RU as a uniaxial structure (see Cronquist, 1981; Trifonova, 1982; Dahlgren et al., 1985; Berry, 2004; Takhtajan, 2009; Ferrari and Oriani, 2017). The single flower is terminal. Our data show that the phyllomes along the RU axis follow the same ontogenetic spiral, with no sign of a cymose pattern. When the phyllomes are initiated, the RU meristem enlarges to become a floral meristem. The developing flower occupies the geometric center of the RU and cannot be assigned to the axil of any neighboring phyllome. Moreover, the sepals alternate with the three uppermost phyllomes, so the position of the uppermost phyllomes determines the sepal positions, demonstrating that both types of phyllome (RU phyllomes and sepals) belong to the same axis. Although the three sepals are initiated simultaneously, they positionally continue the ontogenetic spiral of the phyllomes on the RU axis. A hypothetical situation of a solitary pseudoterminal flower (a lateral flower situated in the axil of the distalmost phyllome of the RU) would expect one of the three sepals to be located on the same radius as the distalmost phyllome. Indeed, trimerous lateral monocot flowers lacking additional phyllomes on the pedicel usually possess a median abaxial outer-whorl perianth member that is located in the same radius as the flower-subtending bract (Eichler, 1875; Remizowa et al., 2013). Therefore, our observation that sepals of Rapateaceae alternate with the three uppermost phyllomes does not support the hypothesis of a pseudoterminal flower. The terminal position of the flower is also shown by the RU vasculature, as the axis is vascularized by two vascular cylinders, the external one supplying the RU phyllomes, and the internal one supplying the flower (Rosa, 2006).

The homologies of the phyllomes situated below the flower are less clear. We did not observe any occasional (even vestigial) branches in their axils, and such branching has not been reported in other studies. Thus, the phyllomes are not the homologs of floral pherophylls (flower-subtending bracts), though they could be interpreted as floral prophylls or bracteoles (Trifonova, 1982; Colella-Franco, 1999; Rosa, 2006). Indeed, the RU phyllomes are initiated in a sequential spiral arrangement, whereas the sepals are whorled and appear simultaneously following a longer plastochron. This development is in accordance with interpretation of the RU phyllomes as prophylls. On the other hand, many monocots possessing such phyllomes on their pedicels display occasional branching in the axils of the floral prophylls. The presence of floral prophylls is a feature that often marks large monocot clades or subclades (Remizowa et al., 2013). In contrast, the vast majority of Poales lack floral prophylls. Other monocots typically possess a single (if any) floral prophyll, rarely two prophylls, whereas the RUs of Rapateaceae have numerous phyllomes. These data tend to contradict their interpretation as floral prophylls; accepting this hypothesis would make Rapateaceae unique among Poales and monocots in general in having numerous floral prophylls.

A third possible interpretation indicates that the RU phyllomes are additional sepals or more likely an epicalyx. Each Rapateaceae RU represents a separate unit that is clearly delimited from the primary inflorescence axis. This delimitation is especially evident in species with stalked RUs. A sepal interpretation is less plausible due to the spiral arrangement of the phyllomes and a characteristic plastochron during RU development, but an epicalyx is more plausible. An epicalyx (or calyculus) is a characteristic feature of Tofieldiaceae (Alismatales) (Eichler, 1875; Remizowa et al., 2006, 2011). This structure consists of three phyllomes alternating with the outer tepals and sometimes separated from the perianth by an internode. In many respects, the calyculus phyllomes behave like an additional perianth whorl and cannot be readily homologized with floral prophylls. Although not equal in number to the sepals and not whorled, the RU phyllomes of Rapateaceae demonstrate some similarity with the calyculus of Tofieldiaceae. However, this hypothesis would also make Rapateaceae unique among Poales and monocots in general in having a calyculus of numerous spirally arranged phyllomes.

The problematic homologies of the phyllomes situated below the flower parallel those reported for Buxaceae and some Ranunculales. In the unisexual flowers of Buxaceae, all of the numerous phyllomes located below the reproductive organs appear superficially similar. However, detailed comparative studies of morphology, phyllotaxis and development allowed a tepal interpretation for the 4–5 uppermost phyllomes and empty flower-subtending bracts for the remainder, some bearing vestigial flowers in their axils (von Balthazar and Endress, 2002). Within Ranunculales, at least three families demonstrate a flower groundplan that is unusual for eudicots, with numerous whorled sepals or tepals, the outermost ones being small and bract-like (Endress, 1995a).

To summarize, the Rapateaceae RU (so-called spikelet) is a uniaxial structure composed of a terminal flower and numerous empty phyllomes below the flower. The occurrence of a terminal flower in an inflorescence unit is an autapomorphic feature of Rapateaceae. Two hypotheses can be proposed to understand RU evolution in Rapateaceae.

(1) The reductional hypothesis would interpret the Rapateaceae RU as a derived condition, in which the lateral flowers of the ancestral spikelet have been eliminated and a terminal flower has appeared to compensate for the loss of the lateral ones (see also Stevenson et al., 1998; Colella-Franco, 1999). In support of this hypothesis, racemose (partial) inflorescences represent the plesiomorphic condition in monocots (Remizowa et al., 2013). The presence or absence of a terminal flower is a labile condition and can be caused by different factors in different angiosperm families (Bull-Hereñu and Claßen-Bockhoff, 2010, 2011a,2011b). Racemose inflorescences of early-divergent monocots (Acorales and Alismatales) show a range of variation in inflorescence tip structure between species of the same family or even between individuals of the same species (Sokoloff et al., 2006; Remizowa et al., 2013).
(2) Alternatively, the Rapateaceae RU could be homologous with a single flower that has undergone a disturbed program of perianth development to produce the extra phyllomes below the flower. In theory, this unusual construction could even have given rise to the more typical spikelets of other Poales. In this scenario, the occurrence of additional phyllomes on the pedicel could have allowed the possibility of further branching in their axils and hence the formation of a spikelet in place of a single flower, the terminal flower having disappeared. This hypothesis is in accordance with the near-basal position of Rapateaceae within Poales in most recent phylogenies (Davis et al., 2004; Givnish et al., 2004, 2010, 2018; McKain et al., 2016), and also with the fact that floral genes are involved in the entire program of spikelet development, at least in grasses (Kellogg, 2015).

### Floral Ontogeny

Floral organ development appears to be more or less uniform in different species of Rapateaceae. Our data on floral development in Rapateoideae showed a similar pattern of floral organ initiation to that of Saxofridericioideae (Ferrari and Oriani, 2017). The perianth and stamens arise as separate primordia. The receptacle is slightly concave and narrow at carpel initiation and the carpel primordia are hidden between the young stamens, a feature that is more pronounced in our material.

Endress (1995b) identified two contrasting developmental patterns in monocots: (1) with tepal and stamen primordia separate from each other and expansion of the floral apex before gynoecium initiation; (2) with the inner tepals (petals) and inner stamens borne on common primordia and delayed expansion of the floral apex after carpel appearance. Rapateaceae flowers demonstrate a mixture of these two patterns – separate primordia of sepals, petals and stamens but a narrow receptacle. Formation of common primordia typically leads to simultaneous initiation of the inner tepals and stamens; exceptions are rare and require careful investigation (Endress, 2010; Remizowa, 2019). Such simultaneous initiation of numerous organs requires more space and meristematic material, leaving only a small area for gynoecium inception. Among Poales, common petal–stamen primordia have been reported for Xyridaceae (Remizowa et al., 2012; Nardi et al., 2021) and Eriocaulaceae (Stützel, 1984; Silva et al., 2016; Sokoloff et al., 2020). Simultaneous organ initiation but with separate primordia is also found in Rapateaceae. In *Saxofridericia aculeata*, all stamens seem to be initiated simultaneously (Ferrari and Oriani, 2017), and in species of *Duckea* (this study) petals and stamens appear without detectable plastochrons. In both genera, simultaneous organ initiation is correlated with delayed floral apex expansion, promoting rapid floral development, and apparently helping to position the carpels prior to postgenital fusion via the available space configuration.

Formation of common primordia and to some degree their detectability is correlated with the geometry of corresponding organs. Common primordia are readily detectable if the organs possess the same tangential width (also at maturity). This is not the case in Rapateaceae, in which the sepals are initiated as broad primordia; as they enlarge, the floral apex takes on a triangular shape with rounded points. The three petals arise in alternisepalous positions from the three points of the triangular apex while the outer stamens are initiated between the petals in antesepalous positions. The width of the outer stamen primordia is much smaller than the sepals, but of almost equal width to the petal primordia. Both the petals and sepals soon become broad. At initiation, the petals are already much broader than the inner stamens. Among other Poales, initiation of the perianth and androecium by separate primordia is also found in Bromeliaceae (Sajo et al., 2004) and Mayacaceae (Oriani and Scatena, 2019). In both of these families, the stamens have narrow bases, and organ initiation is strictly sequential in Bromeliaceae. In Mayacaceae, small antepetalous stamens are absent and three antesepalous stamens appear simultaneously with the petals; the stamen primordia are considerably larger than the petal primordia.

### Gynoecium Construction: Carpel Fusion and Ovule Attachment

The relative roles of postgenital and congenital fusion between carpels in gynoecium formation is a crucial aspect of evolutionary floral morphology. Distinguishing between carpel fusion modes is problematic in anthetic flowers of Rapateaceae, because lines of postgenital fusion become invisible at maturity, so developmental data are the only reliable source to understand gynoecium construction in this family. Unfortunately, developmental material of Rapateaceae is not easy to obtain. We used indirect evidence to distinguish gynoecial zones in the absence of developmental data. The occurrence of heterocarpellous ventral (also termed synventral) bundles is a good marker of congenital carpel fusion. These bundles are shared between two adjacent carpels and lie on septal radii. In the symplicate zone, each heterocarpellous ventral bundle is assumed to represent the two united ventral bundles (see Leinfellner, 1950; for terminology Eyde and Tseng, 1971). Another important character is micropyle orientation, which helps to identify carpel and gynoecial zones. In the ascidiate carpel zone (as well as in the synascidiate gynoecial zone), micropyles are pointed toward the ovary bottom and the ovule itself is inserted vertically. In the plicate carpel zone, micropyles are directed toward the ovary wall and the ovule itself is inserted perpendicular to the ovary and continues the curvature of the carpel margin (Endress, 2019). A combination of synventral bundles and micropyle orientation allows recognition of synascidiate vs. symplicate zones. This criterion works well only in the ovary of Rapateaceae. Ventral bundles in many species examined do not proceed above the placentae and they do not run above the ovary roof in any species. Thus, the role of congenital carpel fusion in the style can only be inferred using developmental data.

Our data on gynoecium structure in Saxofridericioideae–Saxofridericieae are in accordance with a previous detailed report on *Saxofridericia aculeata* (Ferrari and Oriani, 2017), which reported a combination of postgenital and congenital fusion events during gynoecium development. The gynoecium of Saxofridericieae consists of synascidiate, symplicate and asymplicate zones; i.e., both congenital and postgenital fusions between carpels are involved in gynoecium formation. The ovary has a pronounced unilocular region, with the carpel margins protruding into the locule. Numerous ovules are attached in the plicate carpel zone and placentation is parietal (i.e., the ovules are mostly confined to the unilocular region). *Saxofridericia compressa* and *Stegolepis* (Saxofridericioideae–Stegolepideae) differ only by the presence of a hemisymplicate zone. In this region of the ovary, the ovary wall is formed via congenital fusion but the carpel margins bearing the ovules are initially free. In Schoenocephalieae, an asymplicate zone is absent or very short.

Species of Rapateoideae and Monotremoideae demonstrate a similar gynoecium groundplan but differ in the relative lengths of the gynoecial zones and placentation (see also Colella-Franco, 1999; Oriani and Scatena, 2013). Here, the synascidiate zone is fertile, the ovules (one or two per carpel) are attached to the cross-zone, placentation is axial, and the unilocular region of the ovary is very short if present. Our observations have revealed variability of gynoecium development within species of *Duckea*. Although the mature gynoecia appear similar, the length of the congenitally fused regions can differ between flowers belonging to the same inflorescence. In some flowers, the carpels are congenitally united up to the ovary roof, whereas in others the carpels are congenitally united along almost entire their length. Such instability of gynoecium development has not yet been reported for any other monocot species. Among Monotremoideae, *Potarophytum*, and *Monotrema* reveal the presence of postgenital carpel fusion, which is more prominent in *Potarophytum*. *Maschalocephalus* displays exclusively congenitally fused carpels, and an asymplicate zone is absent.

In all species, the carpels are united up to their tips, forming a long and hollow style, though the stylar canal is postgenitally closed at the base in some species. A common style without stylar branches formed by free carpel tips is a rare condition in Poales. Apart from Rapateaceae, a common style is present only in Mayacaceae (De Carvalho et al., 2009; Oriani and Scatena, 2019). The styles in Rapateaceae and Mayacaceae are extremely similar in overall appearance and even in their anatomy, but they differ by their origin. In Mayacaceae, the style is a result of congenital carpel fusion (symplicate zone), whereas in many (but not all) Rapateaceae, the style or its upper part is a product of postgenital carpel fusion (asymplicate zone).

Gynoecium structure and ovule location are widely considered to be conservative characters in monocots, at least at the family level. However, species of Rapateaceae demonstrate contrasting carpel zone fertility and placentation at the level of subfamily. Among other Poales, such variability with respect to ovule insertion, either in the plicate or ascidiate carpel zone, is also found in Xyridaceae (Oriani and Scatena, 2012; Nardi et al., 2021) and Bromeliaceae (Bernardello et al., 1991; Sajo et al., 2004). In Bromeliaceae, it is accompanied by variation in ovary position. Contrasting types of placentation are found in the two largest genera of Juncaceae (Oriani et al., 2012; Shamrov et al., 2012): in *Juncus*, there is a symplicate zone bearing several ovules with parietal placentation, whereas the unilocular gynoecium of *Luzula* possesses only three ovules with basal placentation. In Bromeliaceae and Xyridaceae, the synascidiate zone (if fertile) bears numerous ovules.

Molecular phylogenetic studies tentatively suggest that Rapateoideae is sister to the other two subfamilies of Rapateaceae (Givnish et al., 2000, 2004). Thus, it is possible that a gynoecium with a fertile synascidiate zone and a single pendent ovule per carpel is ancestral in Rapateaceae. Gynoecia of similar construction are widespread among Poales. The occurrence of a single pendent ovule per locule and axial (or basal derived from axial) placentation is shared by Restionaceae, Eriocaulaceae, some Juncaceae, the families of graminid clade with plurilocular gynoecia – Flagellariaceae, Joinvilleaceae, and Ecdeiocoleaceae (Sokoloff et al., 2020 and references therein). Monotremoideae share with Rapateoideae this type of gynoecium construction. If this scenario proves correct, then a fertile plicate carpel zone with numerous ovules should be treated as a derived condition that evolved within the subfamily Saxofridericioideae. A plicate zone allows insertion of more ovules (Doyle and Endress, 2000; Endress and Doyle, 2009) which means increased seed set. Gynoecia with numerous ovules inserted in a plicate carpel zone and often unilocular ovary are characteristic of biotically (insect or bird) pollinated families, including some Xyridaceae (Oriani and Scatena, 2012; Remizowa et al., 2012; Nardi et al., 2021), Mayacaceae (De Carvalho et al., 2009; Oriani and Scatena, 2019), some Bromeliaceae (Bernardello et al., 1991; Sajo et al., 2004), and also some wind-pollinated Juncaceae (Oriani et al., 2012; Shamrov et al., 2012). Xyridaceae and Mayacaceae differ from Rapateaceae by their orthotropous ovules and Bromeliaceae by the presence of septal nectaries and variable ovary position.

### Nectaries

Among Poales, postgenital fusion between the carpels occurs only in Rapateaceae and Bromeliaceae, both biotically pollinated taxa that are placed among the early-divergent lineages of Poales. In Bromeliaceae, postgenital carpel fusion is associated with the formation of an extensive triradiate septal nectary below the ovary locules, associated with the ascidiate carpel zone but opening by three separate slits in the asymplicate zone, where the carpels are partly postgenitally fused (Bernardello et al., 1991; Sajo et al., 2004). The presence of septal (gynopleural) nectaries is one of the unique features of monocots, but this character is not present in Poales except Bromeliaceae [reviewed by Smets et al. (2000), Rudall (2002), and Remizowa et al. (2010)]. Surprisingly, anatomical studies to date have failed to locate septal nectaries in Rapateaceae, even in the bird-pollinated taxa. Slits resembling non-functional septal nectaries appear briefly in development during postgenital fusion between the carpels (Ferrari and Oriani, 2017, this study), but they are indiscernible at maturity. Yet, flowers of many Rapateaceae reportedly produce nectar; for example, flowers of Schoenocephalieae produce copious nectar and demonstrate a bird-pollination syndrome, which is typically associated with a high nectar volume (Rudall et al., 2003; Berry, 2004). Although all Rapateaceae possess trichomes on their floral parts, it seems likely that these structures secrete mucilage rather than nectar. Morphological slits, which are so indicative of septal nectaries, are absent, though we found deep septal grooves in flowers of *Spathanthus*. Here, these slits are non-secretory and formed as a result of late bulging of locules (see also Oriani and Scatena, 2013).

Our study and that of Colella-Franco (1999) indicate a gynoecial non-septal nectary in the bird-pollinated tribe Schoenocephalieae (*Guacamaya, Kunhardtia*, and *Schoenocephalium*), expressed histologically in the epidermis or underlying parenchymatous tissue, mostly below the level of the ovary locules and just above the level of perianth insertion. In all three genera of Schoenocephalieae, the secretion is apparently produced by epidermal cells (Figures 7H,I, 8B,C). Colella-Franco (1999) described and illustrated histological nectaries in *Kunhardtia rhodantha*, with numerous “schizogenic secretory spaces,” though she also reported that these spaces are inconspicuous in *Guacamaya*, in which nectar secretion occurs at the base of the petals. We did not find such secretory parenchyma, but we tentatively confirm a secretory epidermis. Similar gynoecial nectaries have been reported for *Tupistra squalida* (Daumann, 1970), in which the gynoecium consists of congenitally united carpels with no sign of intercarpellary slits; nectary is produced by the epidermis of the ovary, commencing in floral buds 1 day before their opening and lasting for several hours. Another interesting parallel occurs in the monocot family Iridaceae (Rudall et al., 2003), which also displays a range of pollinators and nectary types, including both septal nectaries and perigonal nectaries, in which nectar is secreted from an epidermal region at the base of the perianth tube. Rudall et al. (2003) speculated that perigonal nectaries could have evolved from septal nectaries following temporal or spatial shifts in development (heterochrony or heterotopy) that resulted in their expression in a more distal position on organ primordia. It will be interesting to confirm the phylogenetic placement of Schoenocephalieae to determine whether this tribe evolved from ancestors with septal nectaries.

There is a strong correlation between the presence of septal nectaries and postgenital fusion between carpels. Evolutionary loss of septal nectaries is often associated with a shift from postgenital (or partially postgenital) carpel fusion to congenital fusion, at least in the region where the main part of the nectary was located (e.g., Van Heel, 1988; Rudall, 2002; Remizowa et al., 2010, 2011). The gynoecium of Rapateaceae, with postgenitally united plicate zones, could have evolved from a gynoecium with a superior or semi-inferior ovary with septal nectaries similar that of Bromeliaceae (see also Ferrari and Oriani, 2017). This scenario would involve the loss of the septal nectaries and transition to congenital fusion in the basal portion of gynoecium (this region corresponds to the synascidiate zone in Rapateaceae). Indeed, in the nectar-secreting species of Schoenocephalieae, our study suggests that the secretory function is retained at the gynoecium base (synascidiate zone) but is transferred to the outer epidermis of the ovary base, i.e., to the dorsal carpel side, in contrast to the lateral surfaces in the vast majority of monocots.

In many monocots, apart from the formation of septal nectaries, postgenital fusion facilitates gynoecium closure at the style tip. In Rapateaceae species with an asymplicate zone, a characteristic pattern at the stigma is formed by lines of postgenital fusion between the carpels and postgenitally closed ventral slits. These lines of postgenital fusion are the only ones that remain traceable in mature gynoecia. In species lacking an asymplicate zone, the stigma is open and the gynoecium is sealed by secretion. Interestingly, in species that lack postgenital closure of the style tip (i.e., without an asymplicate zone) the style narrows at its base; the stylar canal in this region becomes triradiate and the style walls contact each other to close the canal lumen.

## Conclusion

Rapateaceae demonstrate a suite of characters that are not found outside this unusual family. The spikelets of Rapateaceae represent a uniaxial system composed of single terminal flower and empty phyllomes below the flower. The occurrence of a terminal flower in an inflorescence unit is an autapomorphic feature of Rapateaceae. The homologies of the terminal flower are clear, and at least two contrasting scenarios can be proposed to explain the differences between spikelet of Rapateaceae and those of other Poales. The nature of the underlying phyllomes is yet to be clarified. All species examined show a similar pattern of floral development with separate primordia of perianth members and stamens. Despite being initiated separately (rather than on common primordia), the petals and all the stamens tend to be initiated simultaneously.

Gynoecia are relatively diverse within Rapateaceae, differing in the relative lengths of the gynoecial zones and their fertility. In Rapateoideae and Monotremoideae, the ovules are inserted in the synascidiate zone and their number is usually one per locule; the ovary is almost trilocular. In all Saxofridericioideae, the ovules are several per locule and inserted in the symplicate or hemisymplicate zone; the ovary possesses a unilocular region. Despite these differences, the ovary is formed via congenital fusion in all subfamilies. Postgenital carpel fusion (asymplicate zone) is involved in formation of the style and in some cases the ovary roof; further developmental study is needed to establish the contribution of postgenital fusion to gynoecium development. Lines of postgenital fusion disappear very early in gynoecium development, making it problematic to interpret gynoecium structure in mature flowers. Postgenital carpel fusion has apparently been entirely lost at least twice in Rapateaceae; an asymplicate zone is absent from gynoecia of Monotremoideae and Schoenocephalieae. In Rapateoideae, an asymplicate zone is usually present, though its length can be variable even at species and individual level. We speculate that a possible adaptive reason to retain postgenital fusion is more secure gynoecium closure at style tip without leaving an unfused carpel margins. The evolutionary loss of postgenital carpel fusion in the ovary region resulted in the elimination of septal nectaries. Most Rapateaceae produce pollen flowers. Nectar secretion apparently occurs only in the bird-pollinated tribe Schoenocephalieae, in which an epidermal nectary is located at the ovary base.

## Data Availability Statement

The original contributions presented in the study are included in the article/Supplementary Material, further inquiries can be directed to the corresponding author.

## Author Contributions

SK involved in sectioning, SEM studies, microscopy, and interpretation. PR involved in conceptualization, microscopy and interpretation, and preparation of the figures. DWS involved in conceptualization, fieldwork, and laboratory work. DDS involved in SEM work, conceptualization, and interpretation. MR prepared the figures and the original draft, and involved in SEM work and microscopy, conceptualization, and interpretation. All authors reviewed drafts of the manuscript, and approved the final draft.

## Conflict of Interest

The authors declare that the research was conducted in the absence of any commercial or financial relationships that could be construed as a potential conflict of interest.

## Publisher’s Note

All claims expressed in this article are solely those of the authors and do not necessarily represent those of their affiliated organizations, or those of the publisher, the editors and the reviewers. Any product that may be evaluated in this article, or claim that may be made by its manufacturer, is not guaranteed or endorsed by the publisher.
