# Supplementary material for: Flower and Spikelet Construction in Rapateaceae (Poales)
Source: Front Plant Sci. 2022 Jan 27;12:813915. doi: 10.3389/fpls.2021.813915 (PMC8829430; doi:10.3389/fpls.2021.813915)
Supplement: Supplementary file 1 [file Data_Sheet_1.docx]

Supplementary Material

# Supplementary Figures


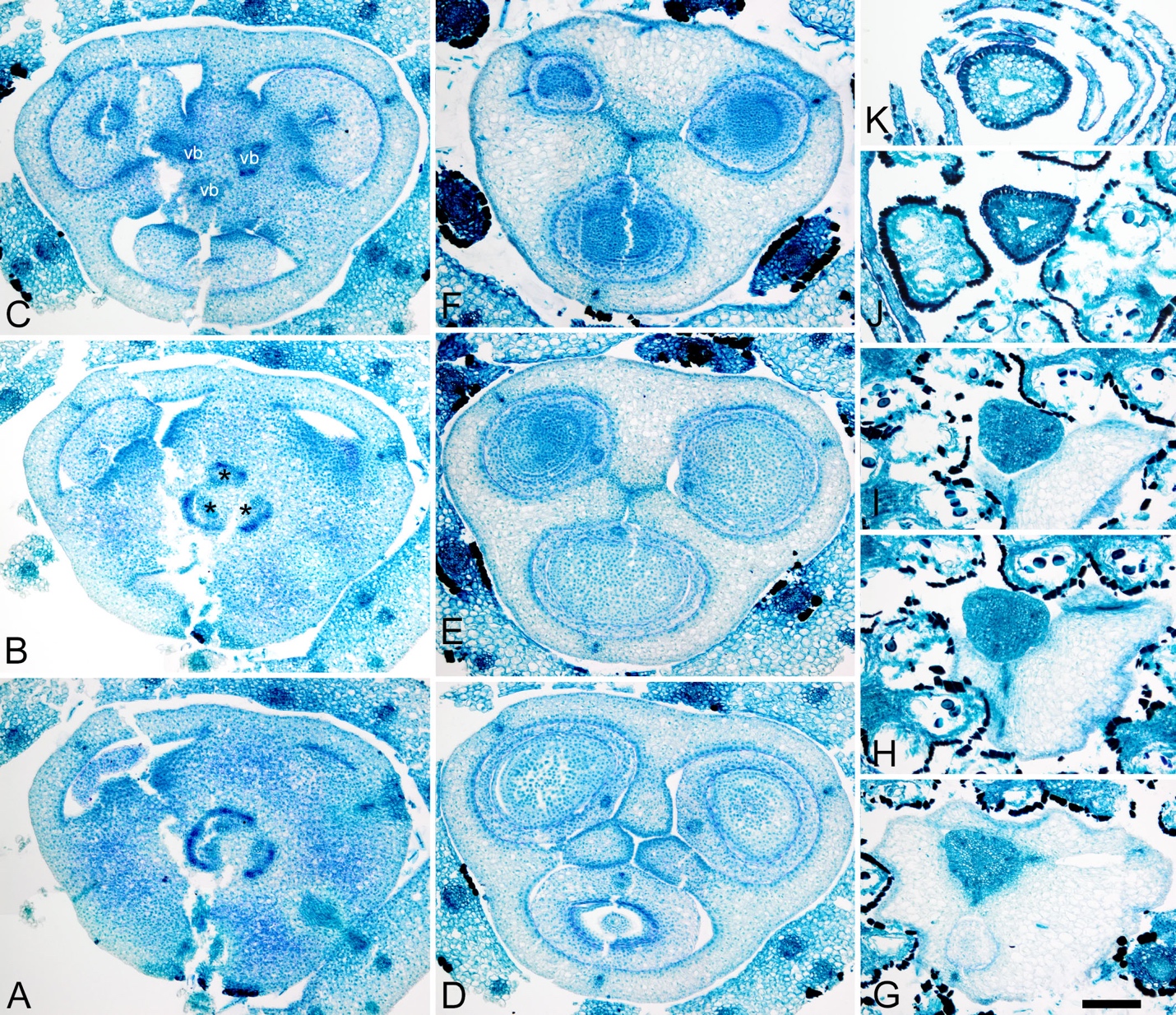


**Supplementary figure 1.** Gynoecium of Rapateoideae. Serial cross-sections through mature gynoecium of *Duckea flava*.

**(A)** Receptacle below ovary. **(B)** Synascidiate zone below ovule insertion, ovary. **(C)** Synascidiate zone, level of ovule insertion. **(D)** Symplicate zone, ovary just above cross-zone. **(E, F)** Symplicate zone, distal part of ovary. **(G)** Ovary roof, symplicate zone. **(H, I)** Style, basal part with no canal visible. **(J)** Style, middle part. **(K)** Style, distal part, asymplicate zone. Vb, homocarpellous ventral bundle, * - heterocarpellous ventral bundle. Scale bars = 200 mkm in all panels.


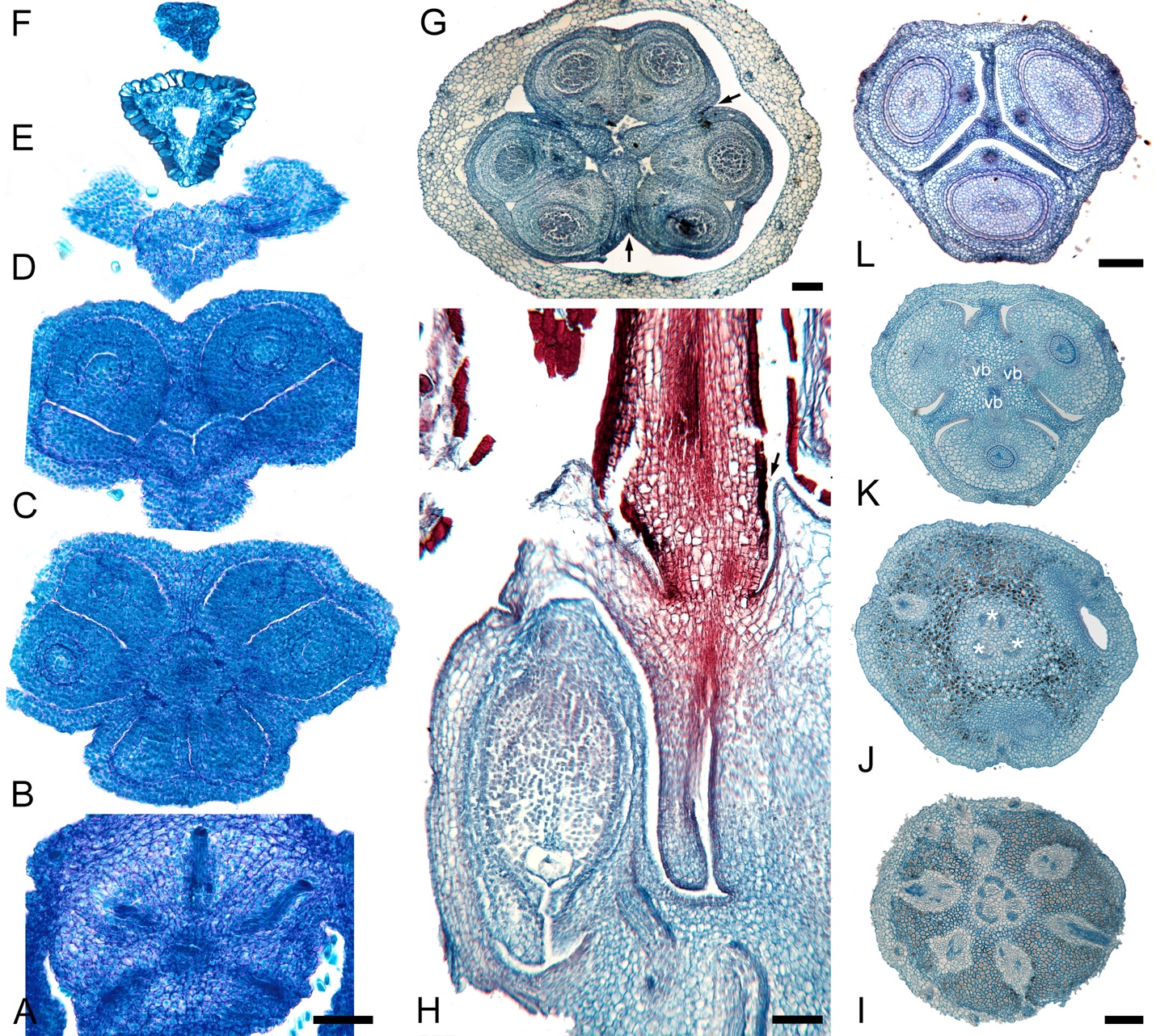


**Supplementary figure 2.** Gynoecium of Rapateoideae. *Spathanthus unilateralis, Duckea flava* and *Rapatea paludosa*.

**(A–F)** Serial cross-sections through young gynoecium of *Spathanthus unilateralis*. **(G)** Section of mature flower of *Spathanthus unilateralis*. **(H)** Longitudinal section of mature flower of *Duckea flava*, **(I–L)** Serial cross-sections of mature flower of *Rapatea paludosa*. **(A)** Receptacle below ovary. **(B)** Synascidiate zone, level ovule insertion, ovary. **(C, G)** Symplicate zone, ovary just above cross-zone. **(D)** Ovary roof, asymplicate zone. **(E)** Style, middle part. **(F)** Stigma. **(I)** Receptacle below ovary. **(J)** Synascidiate zone, level below ovule insertion. **(K)** Synascidiate zone, level of ovule insertion. **(L)** Symplicate zone with postgenitally closed ventral slits, ovary just above cross-zone.

Vb, homocarpellous ventral bundle, * - heterocarpellous ventral bundle. Arrows indicate areas of possible nectar secretion. Scale bars = 100 mkm in panels (A-Н), 20 mkm in panels (I-L).


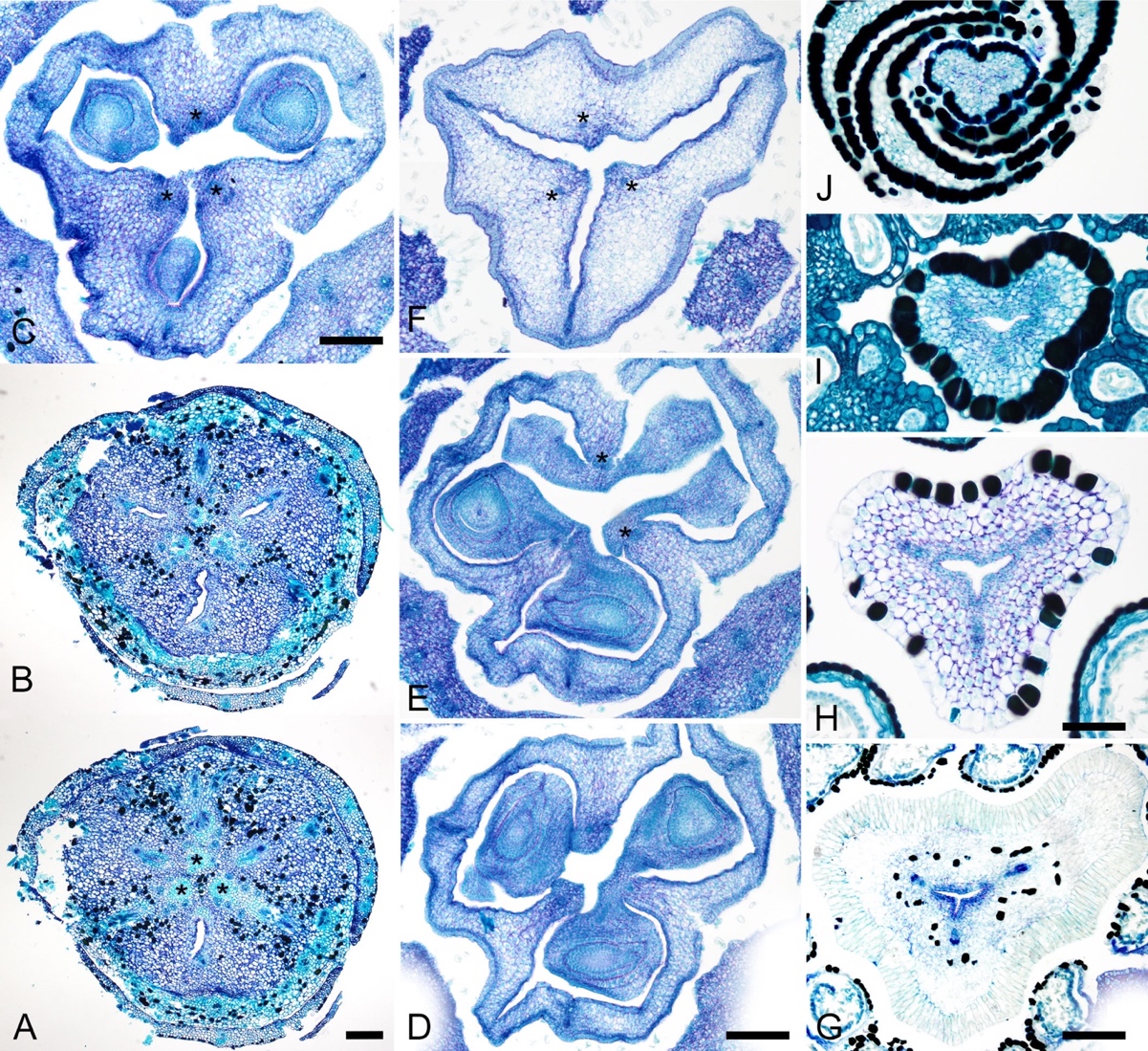


**Supplementary figure 3.** Gynoecium of Saxofridericioideae-Saxofridericieae. Serial cross-sections through mature gynoecium of *Saxofridericia aculeata*.

**(A, B)** sections of young fruit. **(A)** Receptacle below ovary. **(B)** Sterile synascidiate zone. **(C)** Basal portion of symplicate zone below placentae. **(D, E)** Levels of placentae, symplicate zone. **(F)** Symplicate zone above placentae, distal part of ovary. **(G)** Ovary roof. **(H–J)** Style, asymplicate zone, note postgenitally closed gynoecium tip in (J).

* - heterocarpellous ventral bundle. Scale bars = 200 mkm in panels (A-F) and 100 mkm in panels (G-J).


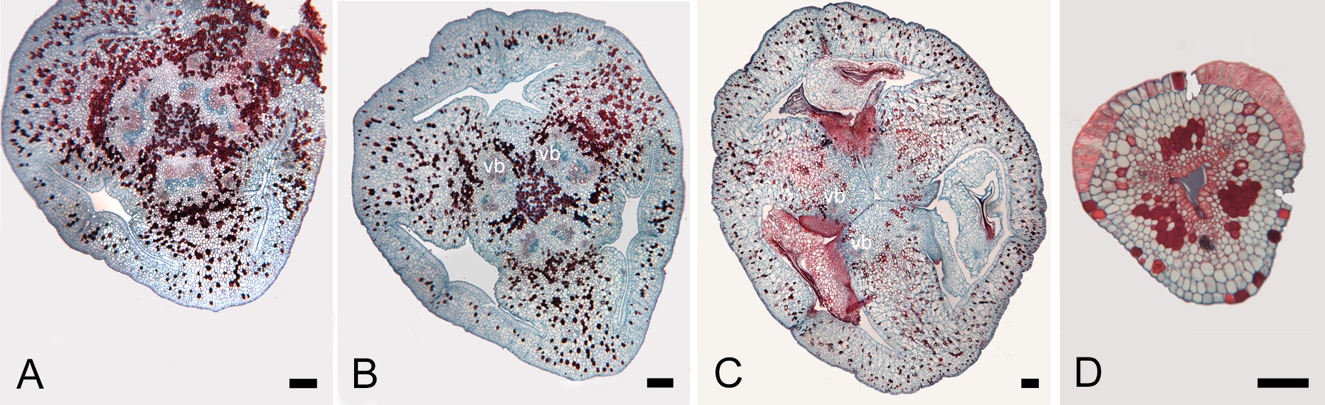


**Supplementary figure 4.** Gynoecium of Saxofridericioideae-Schoenocephalieae. *Guacamaya superba.*

**(A)** Basal part of synascidiate zone with three sets of ventral bundles in septal radii. **(B)** Synascidiate zone. **(C)** Symplicate zone with ovules. **(D)** Style with triradial canal filled with mucilage, symplicate zone.

Vb, homocarpellous ventral bundle. Scale bars = 20 mkm in panels (A–C) and 100 mkm in panels (D).


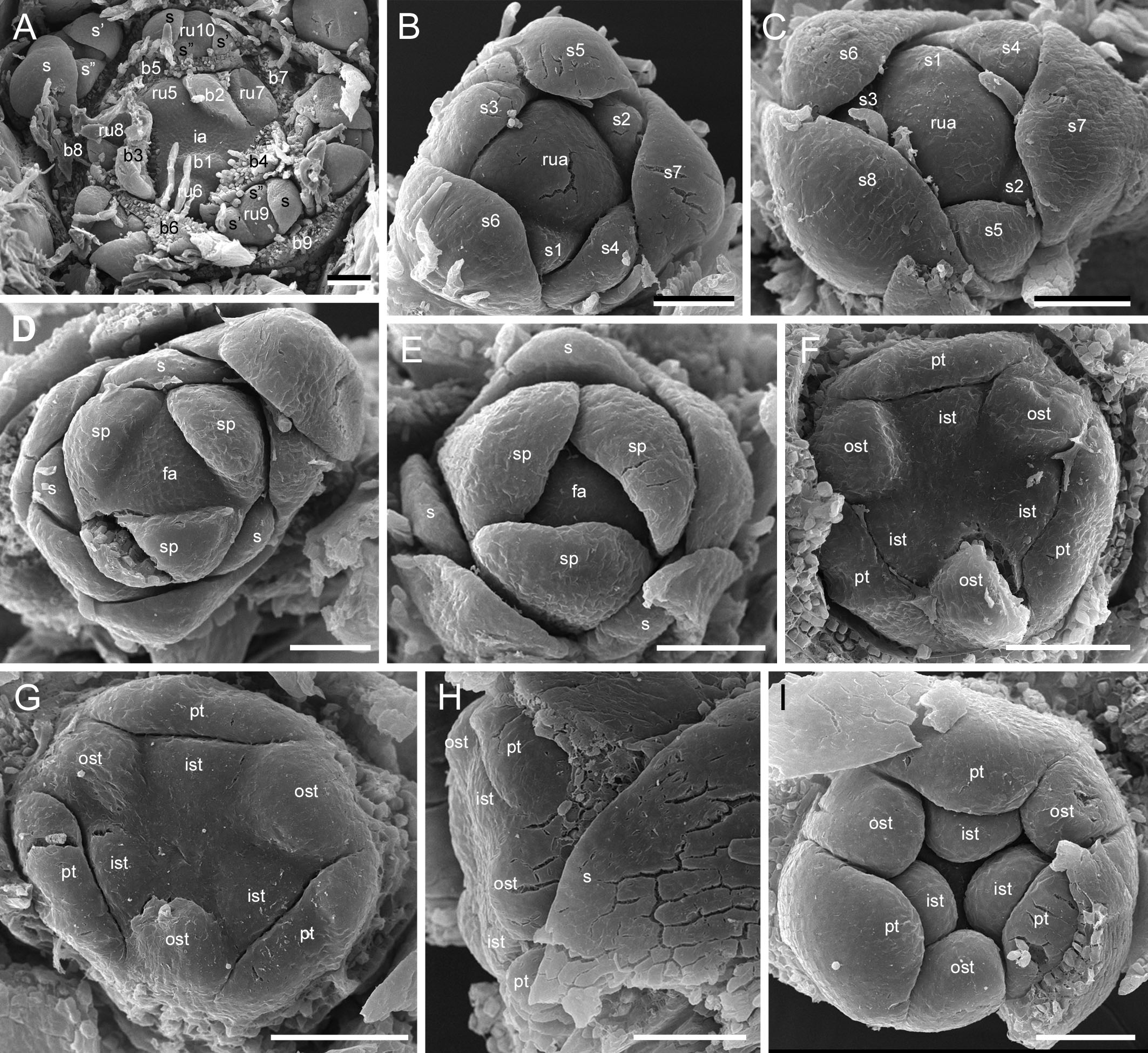
 **Supplementary figure 5.** Flower development in Rapateoideae. *Duckea flava*, early flower development.

**(A)** An inflorescence tip with initiating RUs (‘spikelets’). RUs appear in axils of RU-subtending bracts. The first visible RU is present in the axil of fifth youngest bract. Bracts are numbered starting from inflorescence apex. Numbers of RUs correspond to their subtending bracts. Phyllomes on the RU axis first appear in RU located in the axil of 8^th^ bract. Phyllomes are labelled in the order of their initiation (s – first, s’ – second, s’’ – third) **(B, C)** RUs with spirally initiating phyllomes, numbered starting from RU apex. **(D)** RU terminated by a flower with sepals initiated. Note that sepals alternate with the uppermost phyllomes, which are smaller than sepals. Sepal aestivation is valvate at this stage. **(E)** Sepals grow and their aestivation becomes contort. **(F, G)** Initiation of petals and androecium. Sepals removed. **(H)** Initiation of petals and androecium, side view. **(I)** Young flower at gynoecium initiation, carpels are hidden inside the flower.

B, RU-subtending bract; fa, floral apex; ia, inflorescence apex, ist, inner stamen; ost, outer stamens; pt, petals; rua, RU apex; s, phyllomes on RU axis; sp, sepals. Scale bars = 100 mkm.


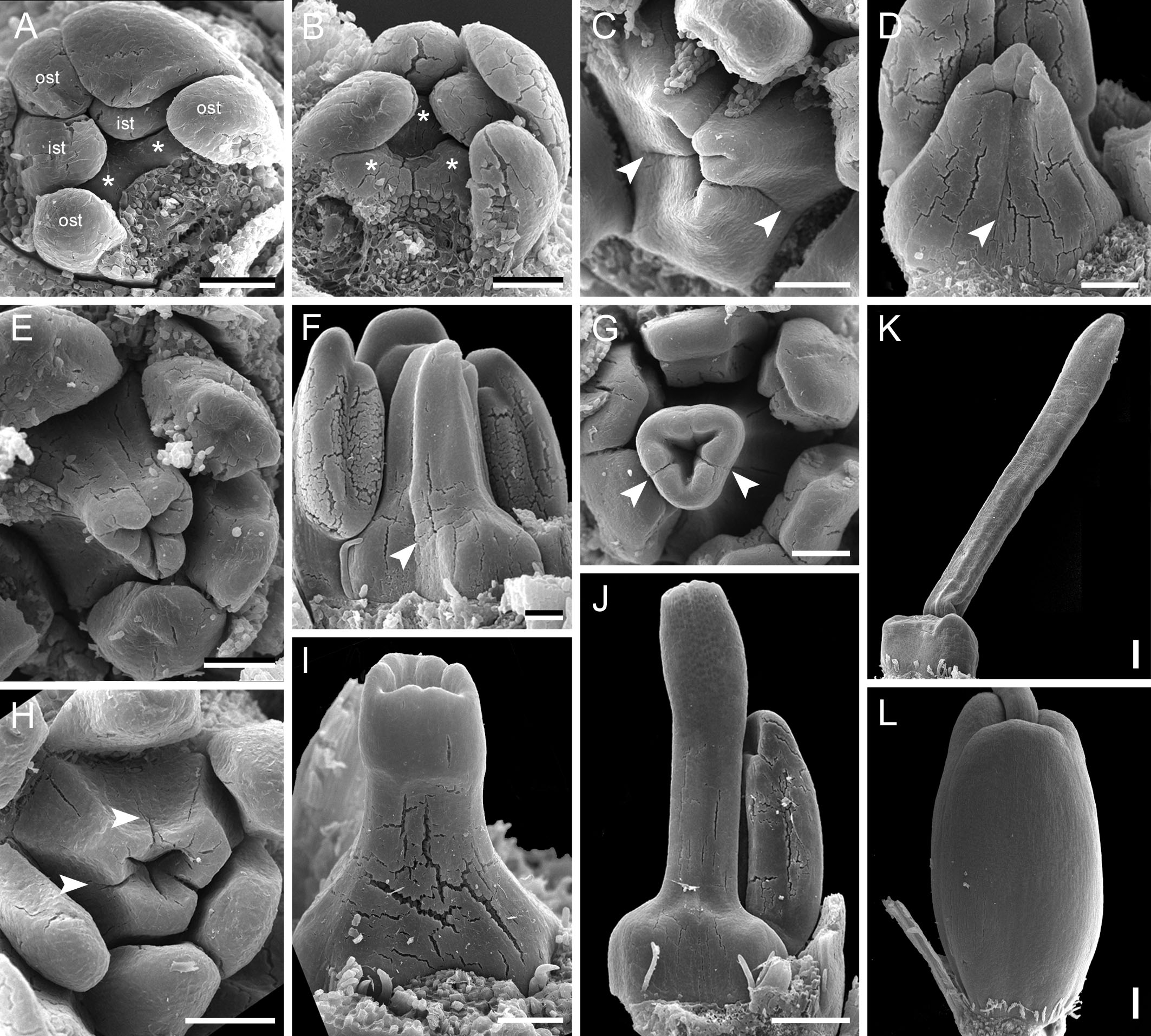


**Supplementary figure 6.** Flower development in Rapateoideae. *Duckea flava*, gynoecium development.

**(A)** Gynoecium initiation as three separate carpel primordia. **(B)** Very young plicate carpels are united via zonal growth. **(C)** Gynoecium with prominent asymplicate zone. Elongation of free plicate carpel parts (asymplicate zone). **(D)** Gynoecium with prominent asymplicate zone. Beginning of postgenital fusion between the carpels. Lines of postgenital fusion are clearly visible. **(E, F)** Gynoecium with prominent asymplicate zone. Boundaries between the carpels become discernible. Ovary is sharply separated from the style. View from above (E), side view (F). **(G)** Stage slightly later than in (E,F). Style tip showing stylar canal. **(H)** Gynoecium with short asymplicate zone. Stage similar to (C). **(I–K)** Progressive stages of style and ovary elongation. Lines of postgenital fusion cannot be traced even in gynoecia with prominent asymplicate zone. **(L)** Mature ovary.

* - carpels; ist, inner stamen; ost, outer stamens; pt, petals. Arrowheads show visible boundaries of postgenital fusion in asymplicate zone. Scale bars = 100 mkm in panels (A–E, G, H) and 300 mkm in panels (F, I, J, K, L).
